# Observability Decomposition-Based Decentralized Kalman Filter and Its Application to Resilient State Estimation under Sensor Attacks

**DOI:** 10.3390/s22186909

**Published:** 2022-09-13

**Authors:** Chanhwa Lee

**Affiliations:** School of Intelligent Mechatronics Engineering, Sejong University, Seoul 05006, Korea; chlee@sejong.ac.kr

**Keywords:** information fusion, decentralized Kalman filter, observability decomposition, attack resilience, secure state estimation, redundant observability, sparse sensor attack

## Abstract

This paper considers a discrete-time linear time invariant system in the presence of Gaussian disturbances/noises and sparse sensor attacks. First, we propose an optimal decentralized multi-sensor information fusion Kalman filter based on the observability decomposition when there is no sensor attack. The proposed decentralized Kalman filter deploys a bank of local observers who utilize their own single sensor information and generate the state estimate for the observable subspace. In the absence of an attack, the state estimate achieves the minimum variance, and the computational process does not suffer from the divergent error covariance matrix. Second, the decentralized Kalman filter method is applied in the presence of sparse sensor attacks as well as Gaussian disturbances/noises. Based on the redundant observability, an attack detection scheme by the $\chi^{2}$ test and a resilient state estimation algorithm by the maximum likelihood decision rule among multiple hypotheses, are presented. The secure state estimation algorithm finally produces a state estimate that is most likely to have minimum variance with an unbiased mean. Simulation results on a motor controlled multiple torsion system are provided to validate the effectiveness of the proposed algorithm.

## 1. Introduction

As control systems operate through network communication and become more complex due to increased connectivity, security against adversarial attacks is becoming more important and receiving attention [1,2,3,4]. In fact, attacks on control systems took place in reality [5,6,7,8], and many studies have been conducted on the security issues of systems whose measurements have been compromised by adversaries because sensors are one of the vulnerable points to malicious attackers in dynamical systems [9,10,11,12,13,14,15].

Among them, the state estimation problem when some of sensors are corrupted by attackers, often called a sparse sensor attack, has been investigated, and several solutions have been recently proposed [10,11,12,13,14,15]. The reference [10] introduces the basic concepts of the secure state estimation problem and formulates it as a non-convex combinatorial optimization problem. The problem is shown to be transformed into a convex optimization problem by using the results developed in the field of compressed sensing [16,17] under additional limiting assumptions. The relationship between this resilient state estimation problem and the notion of strong observability was revealed in [11]. A necessary and sufficient condition for the solvability of this problem is derived in [12,15] with the notion of redundant observability, more specifically, it requires the redundancy of observability twice as much as the sparsity of sensor attacks. A method to alleviate the computational complexity of the logic for finding a combination of non-attacked sensors, is proposed in [13,14]. In [15], the estimator is designed by a set of local observers with only a single sensor, and the decoder uses an error correction algorithm to generate a final state estimate based on the data collected from each local observer.

In addition to sparse sensor attacks, disturbances and noises are considered to enhance the robustness. First, bounded disturbances and noises are considered in [13,15,18], and in particular, the reference [15] explicitly derives the estimation error with the system parameters to provide an analysis of robustness. Second, zero-mean Gaussian white noises and disturbances rather than bounded ones were considered in [19,20,21], and Kalman filters were used to guarantee the state-estimation performance in a probabilistic manner. The reference [19] proposed an estimator with Kalman filters that searches a reliable subset of sensors and operates on the identified subset. A method of combining a secure state estimator and the standard Kalman filter by using the secure state estimator as a pre-filter for the Kalman filter when the set of attacked sensors changes over time, is proposed in [20]. It was shown in [21] that the optimal Kalman estimate can be decomposed into a weighted sum of local estimates, where each estimate uses only a single sensor measurement and that a secure state estimation can be achieved by a convex optimization under some additional assumptions.

This paper considers a general discrete-time linear dynamical system that is corrupted by sparse sensor attacks and Gaussian disturbances/noises. First, we construct local observers on each single sensor and design those local observers with Kalman filters using their own sensor data to cope with Gaussian disturbances/noises. The design of local observers is fully decentralized since it does not utilize any information including Kalman gains or error covariance matrices from other sensors as well as the sensor readings. Furthermore, the local observer’s error covariance is guaranteed not to diverge since it is constructed in the observable subspace based on the observability decomposition, and thus, there is no numerical computational error in practice. Second, a novel information fusion scheme is developed to counteract sparse sensor attacks while maintaining the minimum variance properties. The information fusion center detects the presence of sensor attack in the selected subset of sensors by the $\chi^{2}$ test, which is typically used in the area of fault detection [22,23]. If the $\chi^{2}$ test concludes that there is an attack in the selected subset, a search algorithm is launched to choose a new index set of sensors that is most likely to be unattacked by the multiple hypothesis test. Each hypothesis produces a state estimate with minimum variance, assuming that the index set is attack-free so that each estimate is unbiased. Therefore, the information fusion scheme finally produces a state estimate that is most likely to have the minimum variance and to be unbiased.

Assuming that there exist only Gaussian disturbances/noises without any attacks, a basic information fusion Kalman filter scheme was proposed in [24,25]. The local observers in [24,25] were designed using a Kalman filter for the entire state variable with a single sensor, and a fusion algorithm generates the optimal state estimate with the minimum variance. However, as highlighted in [26], some components of the error covariance may diverge if a single-sensor system is not observable, and this can induce numerical computation problems in practice. This problem can be solved by reducing the target state space to an observable subspace and designing a Kalman filter for the reduced observable subsystem. The idea of decomposing a single-sensor system into the observable subsystem and the unobservable subsystem was proposed in [15] for the secure state estimator design under bounded disturbances/noises, and in [27] for the distributed Luenberger observer design of sensor networks. Hence, adopting this idea and designing the Kalman filter for the observable subsystem, the problem of divergent error covariance does not occur, and we derive the optimal information fusion algorithm even when the size of the local information is different each other.

The contributions of this paper can be summarized as follows:

(1) The proposed algorithm successfully estimates the state variable under sparse sensor attacks as well as Gaussian disturbances/noises. Our algorithm ensures the minimum variance, while [19] simply guarantees that its covariance is no worse than the worst case scenario with high probability;
(2) We only assume that the system is redundant observable, which is known as an equivalent condition for the secure state estimation to be solvable under sparse sensor attacks. Note that [20] requires additional assumptions to reformulate the problem as a convex problem, and further, the combination of Kalman filter and the secure estimator implicitly supposes that the estimation error for the attack signal follows a zero-mean Gaussian distribution, which may not be true when the attack signal is intelligently designed in a coordinated way. The reference [21] needs the system matrix to be nonsingular, and both references [20] and [21] have additional assumptions about the closed-loop system;
(3) The construction of the local observer is completely decentralized, and the overall size of the observer is relatively small. As the combinatorial logic is embedded in the fusion center, we do not have to prepare all possible combinations of observers. Note that [19] does not utilize any decomposition, and thus, it asks for all combinations of observers. The local decomposition presented in [21] is not fully decentralized because the decomposition is performed using the global information of the output matrix and the Kalman gain;
(4) As a by-product obtained during the derivation process, the optimal decentralized information fusion Kalman filter scheme is developed based on the observability decomposition. Compared with the results in [24,25], the proposed scheme does not suffer from the numerical computational errors resulting from the diverging error covariance matrix. The algorithm in this paper guarantees that each error covariance matrix in the local observer converges by the observability decomposition, and this method can also be widely used for the multi-sensor information fusion Kalman filters that do not consider any attacks.

The rest of the paper is organized as follows. The remaining of this section introduces the notation used throughout the paper. The system model and problem formulation are given in Section 2. Section 3 presents the optimal multi-sensor information fusion Kalman filter based on the observability decomposition. We then give the attack detection algorithm by $\chi^{2}$ test and the attack-resilient state estimation scheme by the multiple hypothesis test in Section 4. Finally, simulation results with a servo motor system are given in Section 5, and we provide our concluding remarks in Section 6. The preliminary results of this paper were studied in [28].

*Notation:* Throughout this paper, the following notations are adopted. For a set *S*, the number of elements in the set *S* is denoted by |S|. For a column vector $y\in R^{p}$ and its i-th element $y_{i}$, supp(y) denotes the number of nonzero elements of the vector *y*, that is, $\mathrm{supp}\left(y\right):=\left\{i\in \left[p\right]:y_{i}\neq 0\right\}$ where the symbol [p] is used to represent the subset of natural numbers $\left\{1,2,\cdots ,p\right\}\subset N$. The number of nonzero elements of a vector *y* is defined by the $\ell_{0}$ norm, and it is written as ${∥y∥}_{0}:=\left|\mathrm{supp}\left(y\right)\right|$. We say that the vector *y* is q-sparse if its $\ell_{0}$ norm is less than or equal to q, that is, ${∥y∥}_{0}\leq q$.

For an index set I⊂[p] and a vector $y\in R^{p}$ (or a matrix $C\in R^{p\times n}$), $y_{I}\in R^{\left|I\right|}$ (or $C_{I}\in R^{|I|\times n}$) denotes the vector (or the matrix) obtained from *y* (or *C*) by eliminating all i-th rows such that $i\in I^{c}$. Similarly, for two index sets I,J⊂[p] and a matrix $P\in R^{p\times p}$, $P_{I,J}\in R^{\left|I|\times |J\right|}$ denotes the matrix obtained from *P* by eliminating all i-th rows and all j-th columns such that $i\in I^{c}$ and $j\in J^{c}$.

Let a finite sequence $\left\{\mu_{i}\right\}=\left\{\mu_{1},\mu_{2},\cdots ,\mu_{p}\right\}$ with $\mu =\sum_{i=1}^{p}\mu_{i}$ given. A stacked vector $z={\left[z_{1}^{⊤}\;z_{2}^{⊤}\;\cdots \;z_{p}^{⊤}\right]}^{⊤}\in R^{\mu }$ is said to be partitioned by the sequence $\left\{\mu_{i}\right\}$ if $z_{i}\in R^{\mu_{i}}$ for all i∈[p]. For j∈[p], an index set $I_{j}^{\left\{\mu_{i}\right\}}:=\left\{\left(\sum_{i=1}^{j-1}\mu_{i}\right)+1,\left(\sum_{i=1}^{j-1}\mu_{i}\right)+2,\cdots ,\sum_{i=1}^{j}\mu_{i}\right\}\subset \left[\mu \right]$ represents the j-th partition among total p partitions when a vector $z\in R^{\mu }$ is partitioned by the sequence $\left\{\mu_{i}\right\}$. This notation is extended to a subset J⊂[p] where $I_{J}^{\left\{\mu_{i}\right\}}$ denotes ${⋃}_{j\in J}^{}I_{j}^{\left\{\mu_{i}\right\}}$. A vector $z\in R^{\mu }$ partitioned by the sequence $\left\{\mu_{i}\right\}$, is said to be ($\left\{\mu_{i}\right\}$-stacked) q-sparse if $\left|\left\{j\in \left[p\right]:z_{I_{j}^{\left\{\mu_{i}\right\}}}\neq 0_{\mu_{j}\times 1}\right\}\right|\leq q$.

## 2. System Modeling and Problem Formulation

The plant and the attack model under consideration are presented, and the problem formulation is given in this section.

### 2.1. Plant Modeling with Gaussian Disturbances and Noises

A discrete-time linear time invariant (LTI) system under Gaussian disturbances and noises given by

(1) $$P:\left\{\begin{array}{c} x(k+1)=Ax(k)+Bu(k)+d(k)\\ y(k)=Cx(k)+n(k) \end{array}\right.$$

is considered. In the plant dynamics of (1), $x\in R^{n}$ is the state variable vector, $u\in R^{m}$ is the control input vector, and $y\in R^{p}$ is the sensor output vector. Furthermore, the dynamics is disrupted by the process disturbance $d\in R^{n}$, and the sensors are corrupted by the measurement noise $n\in R^{p}$. There are a total of p sensors that measure the system outputs, and the i-th sensor’s measurement at time *k* is denoted by

$$y_{i}\left(k\right)=c_{i}x\left(k\right)+n_{i}\left(k\right)$$

where $c_{i}$ is the i-th row of the output matrix *C*, which implies that $C={\left[c_{1}^{⊤}\;\;c_{2}^{⊤}\;\;\cdots \;\;c_{p}^{⊤}\right]}^{⊤}$. Here, stochastic assumptions on the disturbance d(k), the noise n(k) and the initial state x(0) of the system (1) are formally stated as follows.

**Assumption** **1.** *The disturbance d(k) and measurement noise n(k) are independent and identically distributed (i.i.d.) white Gaussian process with zero-mean and covariance matrices Q and R, respectively. More specifically*,

$$\begin{array}{cccccc} d(k) & ∼N(0_{n\times 1},\; & & Q), & & \\ n(k) & ∼N(0_{p\times 1},\; & & R), & & \\ E[d(k)] & =0_{n\times 1}, & & E[d\left(k\right)d^{⊤}\;\left(t\right)] & & =Q\delta_{kt},\\ E[n(k)] & =0_{p\times 1}, & & E[n\left(k\right)n^{⊤}\;\left(t\right)] & & =R\delta_{kt},\\ & & & E[n\left(k\right)d^{⊤}\;\left(t\right)] & & =O_{p\times n}, \end{array}$$

*where the symbol E[·] represents the expected value of a random variable and $\delta_{kt}$ is the Kronecker delta function. Furthermore, the initial state x(0) is a Gaussian distributed random variable with the mean ${\bar{x}}_{0}$ and covariance matrix $P_{0}$*,

$$\begin{array}{cc} x(0) & ∼N({\bar{x}}_{0},P_{0}),\\ E[x(0)] & ={\bar{x}}_{0},\;E\left[\left(x\left(0\right)-{\bar{x}}_{0}\right){\left(x\left(0\right)-{\bar{x}}_{0}\right)}^{⊤}\right]=P_{0}, \end{array}$$

*and is independent of d(k) and n(k)*.

### 2.2. Attack Modeling with Sparse Sensor Attacks

Among various attack scenarios [3], we consider false data injection attacks on sensors. Adversarial attackers can inject arbitrary inputs to some (not all) sensors so that a part of the measurements is compromised. Some additive inputs may be induced by cyber or physical tampering with the sensors, or adversaries may penetrate into the communication network on the output side of the plant because those communication links are not secure. In both cases, the attack is characterized by the attack vector $a\in R^{p}$ as in

(2) $$\begin{array}{c} \begin{array}{cc} y^{a}\left(k\right) & =y(k)+a(k)\\ \; & =Cx(k)+n(k)+a(k)\\ \; & =Cx\left(k\right)+n^{a}\left(k\right) \end{array} \end{array}$$

where $y^{a}\in R^{p}$ denotes sensor readings with a potential attack, while $y\in R^{p}$ is the original healthy sensor data affected by the measurement noise only. Similarly, $n^{a}\in R^{p}$ represents the total sensor contamination signal including both the noise *n* and the attack *a*.

Here, it is assumed that the adversaries can compromise only a part of the sensors, not all of them. Assuming that the attacker’s resources are limited, we suppose that the attacker can contaminate up to q out of p measurement outputs. Therefore, a formal condition on the sparsity of the attack vector *a* can be given as follows.

**Assumption** **2.** *The sensor attack vector a(k) is q-sparse for all k≥0, that is, ${∥a\left(k\right)∥}_{0}\leq q,{\;}^{\forall }k\geq 0$. Moreover, it holds that*

$$\left|\left\{i\in \left[p\right]:a_{i}\left(k\right)\neq 0\;\mathrm{for}\;\mathrm{some}\;k\geq 0\right\}\right|\leq q.$$

This assumption tells more than ${∥a\left(k\right)∥}_{0}\leq q$ for all k≥0, in the sense that the compromised sensor channels are not altered for all time. In practice, this may be the case because it takes quite a long time and much effort to infiltrate into a new sensor from a malicious attacker’s point of view. Thus, without loss of generality, it can be assumed that the attack channels remain the same in the long term although it is not revealed to the controller which channels are attacked. However, if the attacked sensor channel changes but does not change frequently, the resilient state estimation scheme to be presented is still applicable. We will simply refer to this assumption as a “q-sparse sensor attack”.

### 2.3. Problem Formulation

For the given discrete-time LTI system (1) under Assumptions 1 and 2, this paper investigates how to design an estimator that can recover the state variable *x* correctly. First, the Gaussian distributed disturbances/noises are handled appropriately, and the optimality in the sense of minimum variance should be recovered. Second, the security against the sparse sensor attack is enhanced, and the attack-resilient estimation with the unbiased state estimate should be achieved. More specifically, this paper considers the problem of proposing a secure and robust state estimation algorithm that generates the estimate that is most likely to have the minimum variance and to be unbiased. In this process, the concept of “redundant observability”, which characterizes the ability of coping with the sparse sensor attack, is utilized to ensure successful state estimation.

The basic condition for the observability of the system (1) with the attack model (2) satisfying Assumption 2, is given in the following assumption. Note that the assumption of “2q redundant observability” is an equivalent condition for the system to be observable under q-sparse sensor attacks ([15], Proposition 2,3,6). Here, the state estimation problem becomes challenging because this redundant observability does not guarantee for the entire states to be recovered with only a single sensor.

**Assumption** **3.** *The system* (1), *or the pair (A,C), is 2q redundant observable. In other words, each pair $\left(A,C_{I}\right)$ is observable for any I⊂[p] satisfying |I|≥p−2q*.

## 3. Optimal Information Fusion Kalman Filter Based on Observability Decomposition

### 3.1. Kalman Observability Decomposition with Single Sensor

Since conventional Luenburger observers or Kalman filters typically have the form of

$$\hat{x}\left(k+1\right)=\left(A-KC\right)\hat{x}\left(k\right)+Bu\left(k\right)+Ky^{a}\left(k\right),$$

the whole state estimates $\hat{x}$ are affected by the single sensor attack signal due to the observer gain *K*. In other words, any single non-zero component of *a* can alter all components of the state estimate $\hat{x}$. Hence, we design a collection of observers where each local observer utilizes only a single sensor information so that an attack signal for one sensor channel only interferes with the corresponding local observer and leaves other local observers unaffected.

Consider a single-output system

(3) $$\begin{array}{c} P_{i}:\left\{\begin{array}{c} x(k+1)=Ax(k)+Bu(k)+d(k)\\ y_{i}^{a}\left(k\right)=c_{i}x\left(k\right)+n_{i}^{a}\left(k\right). \end{array}\right. \end{array}$$

where the i-th component of $y^{a}\left(k\right)$ in (2), $y_{i}^{a}\left(k\right)$, is the output and the dynamics is given by (1). Since the pair $\left(A,c_{i}\right)$ is not necessarily observable, an estimator of the system (3) generally recovers only an (observable) portion of the full state *x*. The Kalman observability decomposition, which clearly describes the observable portion of the system, is now briefly introduced. For the single-output system (3), the observability matrix is written as

(4) $$\begin{array}{c} \begin{array}{c} G_{i}:=\left[\begin{array}{c} c_{i}\\ c_{i}A\\ c_{i}A^{2}\\ \vdots \\ c_{i}A^{n-1} \end{array}\right], \end{array} \end{array}$$

and we denote $\mu_{i}$ as the rank of the observability matrix $G_{i}$. The null space of $G_{i}$, $N(G_{i})$, is the so-called unobservable subspace, and the column range space of $G_{i}^{⊤}$, $R(G_{i}^{⊤})$, is often called the observable subspace.

One can define the similarity transformation as

(5) $$\begin{array}{c} \left[\begin{array}{c} z_{i}\\ w_{i} \end{array}\right]=\left[\begin{array}{c} {Z_{i}}^{⊤}\\ W_{i}^{⊤} \end{array}\right]x \end{array}$$

where $Z_{i}\in R^{n\times \mu_{i}}$ is the matrix whose columns are th orthonormal basis of $R(G_{i}^{⊤})$ and $W_{i}\in R^{n\times (n-\mu_{i})}$ is the matrix whose columns are the orthonormal basis of $N(G_{i})$. Here, the size of those matrices is determined by

$$\mu_{i}=\mathrm{rank}\left(G_{i}\right)=\dim\left(R\left(G_{i}^{⊤}\right)\right)\;\;\;\mathrm{and}\;\;\;n-\mu_{i}=\mathrm{nullity}\left(G_{i}\right)=\dim\left(N\left(G_{i}\right)\right).$$

Note that the observable subspace $R(G_{i}^{⊤})$ is the span of column vectors in $Z_{i}$ and the unobservable subspace $N(G_{i})$ is the span of column vectors in $W_{i}$. Since the matrix $\left[Z_{i}\;\;W_{i}\right]$ is orthogonal, we have

$$\left[\begin{array}{c} {Z_{i}}^{⊤}\\ W_{i}^{⊤} \end{array}\right]\left[\begin{array}{cc} Z_{i} & W_{i} \end{array}\right]=\left[\begin{array}{cc} {Z_{i}}^{⊤}Z_{i} & {Z_{i}}^{⊤}W_{i}\\ W_{i}^{⊤}Z_{i} & W_{i}^{⊤}W_{i} \end{array}\right]=\left[\begin{array}{cc} I_{\mu_{i}\times \mu_{i}} & O_{\mu_{i}\times \left(n-\mu_{i}\right)}\\ O_{\left(n-\mu_{i}\right)\times \mu_{i}} & I_{\left(n-\mu_{i}\right)\times \left(n-\mu_{i}\right)} \end{array}\right].$$

Moreover, because the unobservable subspace is *A*-invariant, any columns of $AW_{i}$ belong to $N\left(G_{i}\right)=R\left(W_{i}\right)$. Therefore, the Kalman observability decomposition of the system (3) is obtained by the transformation (5) as

(6) $$\begin{array}{c} P_{i}^{\prime }:\left\{\begin{array}{c} \left[\begin{array}{c} z_{i}\left(k+1\right)\\ w_{i}\left(k+1\right) \end{array}\right]=\left[\begin{array}{cc} {Z_{i}}^{⊤}AZ_{i} & O_{\mu_{i}\times \left(n-\mu_{i}\right)}\\ W_{i}^{⊤}AZ_{i} & W_{i}^{⊤}AW_{i} \end{array}\right]\left[\begin{array}{c} z_{i}\left(k\right)\\ w_{i}\left(k\right) \end{array}\right]+\left[\begin{array}{c} {Z_{i}}^{⊤}B\\ W_{i}^{⊤}B \end{array}\right]u\left(k\right)+\left[\begin{array}{c} {Z_{i}}^{⊤}\\ W_{i}^{⊤} \end{array}\right]d\left(k\right)\\ y_{i}^{a}\left(k\right)=\left[\begin{array}{cc} c_{i}Z_{i} & 0_{1\times (n-\mu_{i})} \end{array}\right]\left[\begin{array}{c} z_{i}\left(k\right)\\ w_{i}\left(k\right) \end{array}\right]+n_{i}^{a}\left(k\right). \end{array}\right. \end{array}$$

Finally, the state $x\in R^{n}$ is decomposed into the observable sub-state $z_{i}\in R^{\mu_{i}}$ and the unobservable sub-state $w_{i}\in R^{n-\mu_{i}}$. Further, the observable part of (6) can simply be written as

(7) $$\begin{array}{c} P_{i}^{o}:\left\{\begin{array}{c} z_{i}\left(k+1\right)=S_{i}z_{i}\left(k\right)+Z_{i}^{⊤}Bu\left(k\right)+Z_{i}^{⊤}d\left(k\right)\\ y_{i}^{a}\left(k\right)=t_{i}z_{i}\left(k\right)+n_{i}^{a}\left(k\right) \end{array}\right. \end{array}$$

where $S_{i}:=Z_{i}^{⊤}AZ_{i}$ and $t_{i}:=c_{i}Z_{i}$.

### 3.2. Decentralized Multi-Sensor Kalman Filter

Even though the Kalman filter can be applied to unobservable linear systems, the error covariance matrix may not converge in that case. According to ([29], Theorem 26), the detectability of the system is a sufficient condition for the convergence of the error covariance matrix in Kalman filtering. Since detectability is a slightly weaker concept than observability, the results in this paper dealing with observability can be generalized to the concept of detectability with slight modifications. The design of local state estimators for the observable subsystem (7) in the form of Kalman filters using only single sensor information, is derived in this subsection. By its construction, the pair $\left(Z_{i}^{⊤}AZ_{i},c_{i}Z_{i}\right)$, or simply denoted as $\left(S_{i},t_{i}\right)$, is observable, and thus, the error covariance matrix of the Kalman filter designed for the system (7) converges to a positive semidefinite matrix ([29], Theorem 26).

Now, we design a decentralized Kalman filter with each single sensor output, which constitutes the local observer. Then, the design of an information fusion scheme, which collects all the information on state estimates and error covariance matrices from the decentralized Kalman filters, will be discussed in the next subsection. For the simplicity of the derivation, we assume that there are no attacks at this time, that is, a(k)≡0. Thus, $n^{a}\left(k\right)$ and $y^{a}\left(k\right)$ are interpreted as n(k) and y(k), respectively, in this section.

Stochastic assumptions on the disturbance d(k) and the noise n(k) of the system (1) are formally stated in Assumption 1 where the covariance matrix *R* of the measurement noise n(k) is partitioned as

$$R=\left[\begin{array}{cccc} R_{1} & R_{12} & \cdots & R_{1p}\\ R_{21} & R_{2} & \cdots & R_{2p}\\ \vdots & \vdots & \ddots & \vdots \\ R_{p1} & R_{p2} & \cdots & R_{p} \end{array}\right].$$

Finally, the assumption for each measurement noise $n_{i}\left(k\right)$ (which is the same as $n_{i}^{a}\left(k\right)$ in this section) of the system (3) can be written as follows:

$$\begin{array}{cccc} n_{i}\left(k\right) & ∼N(0,R_{i}), & & \\ E[n_{i}\left(k\right)] & =0,\;E[n_{i}\left(k\right)n_{i}^{⊤}\;\left(t\right)] & & =R_{i}\delta_{kt},\\ & \;\;\;\;E[n_{i}\left(k\right)n_{j}^{⊤}\;\left(t\right)] & & =R_{ij}\delta_{kt},\;\mathrm{if}\;i\neq j,\\ & \;\;\;\;E[n_{i}\left(k\right)d^{⊤}\;\left(t\right)] & & =0_{1\times n}. \end{array}$$

The local observer is designed by a Kalman filter for the observable subsystem (7). To this end, let ${\hat{z}}_{i}\left(k|k-1\right)$ be the estimate of $z_{i}\left(k\right)$ based on observations from $y^{a}\left(0\right)$ to $y^{a}\left(k-1\right)$. Similarly, ${\hat{z}}_{i}\left(k|k\right)$ is the estimate of $z_{i}\left(k\right)$ after we process the measurement $y^{a}\left(k\right)$ at time *k*. Following the conventional notations in a Kalman filter, we use the terms $P_{i}\left(k|k-1\right)$ and $P_{i}\left(k|k\right)$ to denote the estimation error covariance of ${\hat{z}}_{i}\left(k|k-1\right)$ and ${\hat{z}}_{i}\left(k|k\right)$, respectively. Thus, We have

(8) $$\begin{array}{c} \begin{array}{cc} P_{i}\left(k|k-1\right) & =E[\left({\hat{z}}_{i}\left(k|k-1\right)-z_{i}\left(k\right)\right){\left({\hat{z}}_{i}\left(k|k-1\right)-z_{i}\left(k\right)\right)}^{⊤}],\\ P_{i}\left(k|k\right) & =E[\left({\hat{z}}_{i}\left(k|k\right)-z_{i}\left(k\right)\right){\left({\hat{z}}_{i}\left(k|k\right)-z_{i}\left(k\right)\right)}^{⊤}]. \end{array} \end{array}$$

Then, the Kalman filter has the following form of

(9) $$\begin{array}{cc} O_{i}:\; & {\hat{z}}_{i}\left(k+1|k+1\right)\\ =\; & S_{i}{\hat{z}}_{i}\left(k|k\right)+Z_{i}^{⊤}Bu\left(k\right)+K_{i}\left(k+1\right)\left(y_{i}^{a}\left(k+1\right)-t_{i}\left(S_{i}{\hat{z}}_{i}\left(k|k\right)+Z_{i}^{⊤}Bu\left(k\right)\right)\right)\\ =\; & \left(I-K_{i}\left(k+1\right)t_{i}\right)\left(S_{i}{\hat{z}}_{i}\left(k|k\right)+Z_{i}^{⊤}Bu\left(k\right)\right)+K_{i}\left(k+1\right)y_{i}^{a}\left(k+1\right), \end{array}$$

where

(10a) $$\begin{array}{c} \begin{array}{cc} {\hat{z}}_{i}\left(k+1|k+1\right) & ={\hat{z}}_{i}\left(k+1|k\right)+K_{i}\left(k+1\right)\left(y_{i}^{a}\left(k+1\right)-t_{i}{\hat{z}}_{i}\left(k+1|k\right)\right) \end{array} \end{array}$$

(10b) $$\begin{array}{c} \begin{array}{cc} {\hat{z}}_{i}\left(k+1|k\right) & =S_{i}{\hat{z}}_{i}\left(k|k\right)+Z_{i}^{⊤}Bu\left(k\right) \end{array} \end{array}$$

(10c) $$\begin{array}{c} \begin{array}{cc} K_{i}\left(k+1\right) & =P_{i}\left(k+1|k\right)t_{i}^{⊤}{\left(t_{i}P_{i}\left(k+1|k\right)t_{i}^{⊤}+R_{i}\right)}^{-1} \end{array} \end{array}$$

(10d) $$\begin{array}{c} \begin{array}{cc} P_{i}\left(k+1|k\right) & =S_{i}P_{i}\left(k|k\right)S_{i}^{⊤}+Z_{i}^{⊤}QZ_{i} \end{array} \end{array}$$

(10e) $$\begin{array}{c} \begin{array}{cc} P_{i}\left(k+1|k+1\right) & =\left(I-K_{i}\left(k+1\right)t_{i}\right)P_{i}\left(k+1|k\right) \end{array} \end{array}$$

with initial value of

$$\begin{array}{c} \begin{array}{c} {\hat{z}}_{i}\left(0|0\right)=Z_{i}^{⊤}{\bar{x}}_{0},\;P_{i}\left(0|0\right)=Z_{i}^{⊤}P_{0}Z_{i}. \end{array} \end{array}$$

The above Equations (10) describe the recursive form of how the state estimate ${\hat{z}}_{i}$, the Kalman gain $K_{i}$, and the error covariance matrix $P_{i}$ evolve. The error covariance $P_{i}$ of the i-th local observer defined in (8), is governed by Equations (10d) and (10e), which ensure that the covariance matrix $P_{i}\left(k|k\right)$ can be calculated by the following recursive form:

(11) $$\begin{array}{cc} L_{i}:\;P_{i}\left(k+1|k+1\right) & =\left(I-K_{i}\left(k+1\right)t_{i}\right)\left(S_{i}P_{i}\left(k|k\right)S_{i}^{⊤}+Z_{i}^{⊤}QZ_{i}\right) \end{array}$$

with the initial value of

$$P_{i}\left(0|0\right)=Z_{i}^{⊤}P_{0}Z_{i}.$$

Similarly, the error cross covariance $P_{ij}$ of the i-th and j-th local observers can be defined by

(12) $$\begin{array}{c} \begin{array}{cc} P_{ij}\left(k|k-1\right) & =E[\left({\hat{z}}_{i}\left(k|k-1\right)-z_{i}\left(k\right)\right){\left({\hat{z}}_{j}\left(k|k-1\right)-z_{j}\left(k\right)\right)}^{⊤}],\\ P_{ij}\left(k|k\right) & =E[\left({\hat{z}}_{i}\left(k|k\right)-z_{i}\left(k\right)\right){\left({\hat{z}}_{j}\left(k|k\right)-z_{j}\left(k\right)\right)}^{⊤}], \end{array} \end{array}$$

and the recursive formula for $P_{ij}$ is derived here. To this end, define the estimation error

(13) $$\begin{array}{c} \begin{array}{cc} {\tilde{z}}_{i}\left(k+1|k\right) & :={\hat{z}}_{i}\left(k+1|k\right)-z_{i}\left(k+1\right)\\ {\tilde{z}}_{i}\left(k+1|k+1\right) & :={\hat{z}}_{i}\left(k+1|k+1\right)-z_{i}\left(k+1\right), \end{array} \end{array}$$

and we have that

(14a) $$\begin{array}{cc} {\tilde{z}}_{i}\left(k+1|k\right) & =\left(S_{i}{\hat{z}}_{i}\left(k|k\right)+Z_{i}^{⊤}Bu\left(k\right)\right)-\left(S_{i}z_{i}\left(k\right)+Z_{i}^{⊤}Bu\left(k\right)+Z_{i}^{⊤}d\left(k\right)\right)\\ \; & =S_{i}{\tilde{z}}_{i}\left(k|k\right)-Z_{i}^{⊤}d\left(k\right) \end{array}$$

(14b) $$\begin{array}{cc} {\tilde{z}}_{i}\left(k+1|k+1\right) & =\left({\hat{z}}_{i}\left(k+1|k\right)+K_{i}\left(k+1\right)\left(y_{i}^{a}\left(k+1\right)-t_{i}{\hat{z}}_{i}\left(k+1|k\right)\right)\right)-z_{i}\left(k+1\right)\\ \; & =\left(I-K_{i}\left(k+1\right)t_{i}\right){\tilde{z}}_{i}\left(k+1|k\right)+K_{i}\left(k+1\right)n_{i}^{a}\left(k+1\right). \end{array}$$

By substituting (14a) into (14b), the dynamics of the error ${\tilde{z}}_{i}\left(k|k\right)$ is obtained as

(15) $$\begin{array}{c} \begin{array}{cc} F_{i}:\;{\tilde{z}}_{i}\left(k+1|k+1\right) & =\left(I-K_{i}\left(k+1\right)t_{i}\right)S_{i}{\tilde{z}}_{i}\left(k|k\right)-\left(I-K_{i}\left(k+1\right)t_{i}\right)Z_{i}^{⊤}d\left(k\right)\\ \; & \;\;\;\;+K_{i}\left(k+1\right)n_{i}^{a}\left(k+1\right). \end{array} \end{array}$$

The errors ${\tilde{z}}_{i}\left(k|k\right)$ and ${\tilde{z}}_{j}\left(k|k\right)$ for i≠j may be correlated; thus, by using (15), the error cross covariance between ${\tilde{z}}_{i}\left(k|k\right)$ and ${\tilde{z}}_{j}\left(k|k\right)$ can be computed recursively. From the recursive form of (15), note that ${\tilde{z}}_{i}\left(k|k\right)$ is a linear combination of elements in

(16) $$\begin{array}{c} \{{\tilde{z}}_{i}\left(0|0\right),d\left(0\right),\cdots ,d\left(k-1\right),n_{i}^{a}\left(0\right),\cdots ,n_{i}^{a}\left(k\right)\}. \end{array}$$

Therefore, by Assumption 1, we have (i) $n_{i}^{a}\left(k+1\right)$ and d(k) are orthogonal, (ii) ${\tilde{z}}_{i}\left(k|k\right)$ and d(k) are orthogonal, and (iii) ${\tilde{z}}_{i}\left(k|k\right)$ and $n_{j}^{a}\left(k+1\right)$ are orthogonal. Using these facts, one can derive the recursive form of the error cross covariance between ${\tilde{z}}_{i}\left(k|k\right)$ and ${\tilde{z}}_{j}\left(k|k\right)$ as follows:

(17) $$\begin{array}{cc} L_{ij}:\; & P_{ij}\left(k+1|k+1\right)=E\left[{\tilde{z}}_{i}\left(k+1|k+1\right){\tilde{z}}_{j}^{⊤}\left(k+1|k+1\right)\right]\\ = & \;\left(I-K_{i}\left(k+1\right)t_{i}\right)\left(S_{i}E\left[{\tilde{z}}_{i}\left(k|k\right){\tilde{z}}_{j}^{⊤}\left(k|k\right)\right]S_{j}^{⊤}+Z_{i}^{⊤}QZ_{j}\right){\left(I-K_{j}\left(k+1\right)t_{j}\right)}^{⊤}\\ \; & +K_{i}\left(k+1\right)E\left[n_{i}^{a}\left(k+1\right){n_{j}^{a}}^{⊤}\left(k+1\right)\right]K_{j}^{⊤}\left(k+1\right)\\ = & \;\left(I-K_{i}\left(k+1\right)t_{i}\right)\left(S_{i}P_{ij}\left(k|k\right)S_{j}^{⊤}+Z_{i}^{⊤}QZ_{j}\right){\left(I-K_{j}\left(k+1\right)t_{j}\right)}^{⊤}\\ \; & +K_{i}\left(k+1\right)R_{ij}K_{j}^{⊤}\left(k+1\right), \end{array}$$

with the initial value of

$$P_{ij}\left(0|0\right)=Z_{i}^{⊤}P_{0}Z_{j}.$$

### 3.3. Optimal Information Fusion Based on Observability Decomposition

Based on the equivalence $Z_{i}^{⊤}x=z_{i}$ in (5) and the definition ${\tilde{z}}_{i}={\hat{z}}_{i}-z_{i}$ in (13), we have

(18) $${\hat{z}}_{i}=z_{i}+{\tilde{z}}_{i}=Z_{i}^{⊤}x+{\tilde{z}}_{i}.$$

Stacking Equations (18) for all i∈[p] leads to the following equation of

(19) $$\left[\begin{array}{c} {\hat{z}}_{1}\left(k|k\right)\\ \vdots \\ {\hat{z}}_{p}\left(k|k\right) \end{array}\right]=\left[\begin{array}{c} z_{1}\left(k\right)\\ \vdots \\ z_{p}\left(k\right) \end{array}\right]+\left[\begin{array}{c} {\tilde{z}}_{1}\left(k|k\right)\\ \vdots \\ {\tilde{z}}_{p}\left(k|k\right) \end{array}\right]=\left[\begin{array}{c} {Z_{1}}^{⊤}\\ \vdots \\ {Z_{p}}^{⊤} \end{array}\right]x\left(k\right)+\left[\begin{array}{c} {\tilde{z}}_{1}\left(k|k\right)\\ \vdots \\ {\tilde{z}}_{p}\left(k|k\right) \end{array}\right].$$

Finally, (19) is written in a compact form as

(20) $$\begin{array}{c} \hat{z}\left(k|k\right)=\Phi x\left(k\right)+\tilde{z}\left(k|k\right)=\Phi x\left(k\right)+v^{a}\left(k\right)\in R^{\mu }, \end{array}$$

where the matrix

(21) $$\Phi :=\left[\begin{array}{c} {Z_{1}}^{⊤}\\ \vdots \\ {Z_{p}}^{⊤} \end{array}\right]\in R^{\mu \times n}$$

is composed of the similarity transformation matrices $Z_{i}$’s and $v^{a}\left(k\right)$ is used for a simple notation of $\tilde{z}\left(k|k\right)$. In Equation (20),

$$\mu :=\sum_{i=1}^{p}\mu_{i}$$

denotes the size of the stacked vector.

**Figure 1:**
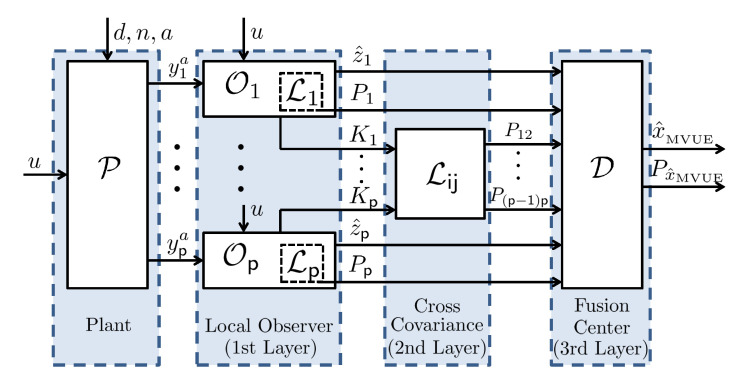
Structure of decentralized multi-sensor information fusion Kalman filter.

It should be noted that all the information in (20) except the actual state x(k), are known or accessible to us. In Section 3.1, the matrix Φ is generated from the orthonormal basis of the observable subspace $R(G_{i}^{⊤})$ where $G_{i}$ is the observability matrix given by (4). In Section 3.2, each local observer $O_{i}$ in (9) provides the state estimate ${\hat{z}}_{i}$ for the observable sub-state $z_{i}$. Now, the stochastic properties of the last term

$$v^{a}\left(k\right)=\tilde{z}\left(k|k\right)=\left[\begin{array}{c} {\tilde{z}}_{1}\left(k|k\right)\\ {\tilde{z}}_{2}\left(k|k\right)\\ \vdots \\ {\tilde{z}}_{p}\left(k|k\right) \end{array}\right]$$

are analyzed. First, its mean is zero because ${\tilde{z}}_{i}\left(k|k\right)$ is a linear combination of elements in (16) by the Formula (15), and Assumption 1 ensures that every component in (16) has a zero mean. Second, the covariance matrix of $v^{a}\left(k\right)$ can be obtained since the error covariance matrix $P_{i}$ is computed by each local observer $L_{i}$ in (11), and the error cross covariance matrix $P_{ij}$ is generated by the second layer of the multi-sensor Kalman filter $L_{ij}$ in (17) with collected information from local observers (see Figure 1 for the structure of the proposed Kalman filter). In summary, we have

(22) $$v^{a}\left(k\right)∼N\left(0_{\mu \times 1},P\left(k|k\right)\right),$$

where

(23) $$P\left(k|k\right)\;=\;\left[\begin{array}{cccc} P_{1}\left(k|k\right) & P_{12}\left(k|k\right) & \cdots & P_{1p}\left(k|k\right)\\ P_{21}\left(k|k\right) & P_{2}\left(k|k\right) & \cdots & P_{2p}\left(k|k\right)\\ \vdots & \vdots & \ddots & \vdots \\ P_{p1}\left(k|k\right) & P_{p2}\left(k|k\right) & \cdots & P_{p}\left(k|k\right) \end{array}\right],$$

which can be recursively computed by (11) and (17). Finally, Equation (20) depicts a linear model with the measured data vector $\hat{z}$, the known matrix Φ, the noise vector $v^{a}$ with a zero-mean Gaussian distribution, and the unknown vector *x* to be estimated.

Based on the statistical estimation and detection theory [30,31], an elaborate derivation process to recover the optimal estimate of *x* in (20), is now presented. The minimum variance unbiased estimator (MVUE) for the data model (20) with $v^{a}$ satisfying $v^{a}∼N\left(0_{\mu \times 1},P\right)$ is introduced as follows.

**Theorem** **1** ([30], Theorem 4.2)**.** *For the measurement $\hat{z}=\Phi x+v^{a}\in R^{\mu }$ with $x\in R^{n}$ and $v^{a}\in R^{\mu }$ such that $v^{a}∼N\left(0_{\mu \times 1},P\right)$ for some P>0, the minimum variance unbiased estimator (MVUE) of x is*

(24) $$D:\;{\hat{x}}_{\mathrm{MVUE}}={\left(\Phi^{⊤}P^{-1}\Phi \right)}^{-1}\Phi^{⊤}P^{-1}\hat{z}$$

*and the corresponding covariance matrix of ${\hat{x}}_{\mathrm{MVUE}}$ is*

(25) $$P_{{\hat{x}}_{\mathrm{MVUE}}}={\left(\Phi^{⊤}P^{-1}\Phi \right)}^{-1},$$

*which achieves the minimum covariance in the sense that $P_{{\hat{x}}_{\mathrm{MVUE}}}\leq P_{\hat{x}}$ for any type of estimator $\hat{x}$*.

**Proof.**  The results directly follows from the Gauss–Markov Theorem ([30], Theorem 6.1). However, we provide a direct proof for the readers convenience, and it follows the procedure in the proof of ([24], Theorem 1) or ([25], Theorem 1). We introduce a linear unbiased estimator

$$\hat{x}=\Omega \hat{z}$$

and, from the unbiased assumption, it follows that

$$E\left[\hat{x}\right]=E\left[\Omega \hat{z}\right]=\Omega E\left[\Phi x+v^{a}\right]=\Omega \Phi E\left[x\right]=E\left[x\right].$$

Thus, we have

(26) $$\Omega \Phi =I_{n\times n}.$$

Let the covariance matrix of the estimation error $\tilde{x}:=\hat{x}-x$ be $P_{x}$. Then, the estimation error $\tilde{x}$ is obtained that

$$\tilde{x}=\hat{x}-x=\Omega \hat{z}-x=\Omega \hat{z}-\Omega \Phi x=\Omega \left(\hat{z}-\Phi x\right)=\Omega v^{a},$$

and the covariance matrix $P_{x}$ can be computed as

$$P_{x}=E\left[\tilde{x}{\tilde{x}}^{⊤}\right]=E\left[\Omega v^{a}{v^{a}}^{⊤}\Omega^{⊤}\right]=\Omega E\left[v^{a}{v^{a}}^{⊤}\right]\Omega^{⊤}=\Omega P\Omega^{⊤}.$$

In order to find the minimum variance estimator, set the trace of the covariance matrix $P_{x}$ as the performance index

$$J:=\mathrm{tr}\left(P_{x}\right)=\mathrm{tr}\left(\Omega P\Omega^{⊤}\right).$$

The Lagrangian [32] associated with *J* becomes

$$L=J+2\mathrm{tr}\left(\Lambda \left(\Omega \Phi -I_{n\times n}\right)\right)$$

where $\Lambda \in R^{n\times n}$ is a matrix representing the Lagrange multipliers. By solving

$$\frac{\partial L}{\partial \Omega }=O_{n\times \mu },$$

we have

(27) $$\Omega P+\Lambda^{⊤}\Phi^{⊤}=O_{n\times \mu }.$$

Combining (26) and (27) results in the following equation of

$$\left[\begin{array}{cc} \Omega & \Lambda^{⊤} \end{array}\right]\left[\begin{array}{cc} P & \Phi \\ \Phi^{⊤} & O_{n\times n} \end{array}\right]=\left[\begin{array}{cc} O_{n\times \mu } & I_{n\times n} \end{array}\right].$$

Therefore, the matrix inversion lemma ([33], Section 2.3) yields the solution as

$$\left[\begin{array}{cc} \Omega & \Lambda^{⊤} \end{array}\right]=\left[\begin{array}{cc} O_{n\times \mu } & I_{n\times n} \end{array}\right]{\left[\begin{array}{cc} P & \Phi \\ \Phi^{⊤} & O_{n\times n} \end{array}\right]}^{-1}=\left[\begin{array}{cc} {\left(\Phi^{⊤}P^{-1}\Phi \right)}^{-1}\Phi^{⊤}P^{-1} & -{\left(\Phi^{⊤}P^{-1}\Phi \right)}^{-1} \end{array}\right].$$

Thus, we have $\Omega ={\left(\Phi^{⊤}P^{-1}\Phi \right)}^{-1}\Phi^{⊤}P^{-1}$. Finally, the MVUE of *x* in (24), is obtained from ${\hat{x}}_{\mathrm{MVUE}}=\Omega \hat{z}={\left(\Phi^{⊤}P^{-1}\Phi \right)}^{-1}\Phi^{⊤}P^{-1}\hat{z}$, and the corresponding covariance matrix in (25) is computed by $P_{{\hat{x}}_{\mathrm{MVUE}}}=\Omega P\Omega^{⊤}={\left(\Phi^{⊤}P^{-1}\Phi \right)}^{-1}$.    □

Theorem 1 explains how the optimal estimate is computed. The information fusion center D calculates the MVUE by (24) and its covariance by (25). In summary, the whole structure of the decentralized multi-sensor information fusion Kalman filter is shown in Figure 1. The first layer is composed of the local observer $O_{i}$, which generates the estimate ${\hat{z}}_{i}$ and the Kalman gains $K_{i}$ as given in (9) and (10). A part of the local observer $O_{i}$, denoted as $L_{i}$, provides the error covariance matrix $P_{i}$. The second layer $L_{ij}$ collects the Kalman gain $K_{i}$’s from the first layer and gives the error cross covariance matrix $P_{ij}$ by (17). Finally, the third layer operates as an optimal information fusion center D as described in Theorem 1 and computes the optimal estimate with the minimum covariance.

**Remark** **1.** *Note that Gauss–Markov Theorem ([30], Theorem 6.1) gives the best linear unbiased estimator (BLUE) for the measurement $\hat{z}=\Phi x+v^{a}$ where $v^{a}$ is a random variable, whose probability density function (PDF) is not restricted to a Gaussian distribution, with a zero mean and covariance P. Since the BLUE is also the MVUE for Gaussian data, the results of Theorem 1 also follow directly from the Gauss–Markov Theorem. The state estimate ${\hat{x}}_{\mathrm{MVUE}}$ given in Theorem 1 is the optimal estimate since it achieves the minimum variance with an unbiased mean. A special case of Theorem 1 is considered in ([24], Theorem 1) and ([25], Theorem 1) for an information fusion scheme; however, the scheme in [24,25] may not be successful for a system whose local systems with a single sensor are not observable because the covariance matrix P could diverge in that case, whereas the covariance matrix P does not diverge in our scheme due to the Kalman observability decomposition.*

## 4. Attack Resilient and Secure State Estimation by Decentralized Kalman Filter

### 4.1. Effect of Sparse Sensor Attack on Information Fusion Kalman FIlter

In the previous section, we assumed that all sensors were attack-free, that is, a(k)≡0. Hence, $n_{i}^{a}\left(k\right)$ and $y_{i}^{a}\left(k\right)$ in (3) and (7) were regarded as non-attacked noise $n_{i}\left(k\right)$ and output $y_{i}\left(k\right)$, respectively. The effects of a sparse sensor attack satisfying Assumption 2 on the information fusion Kalman filter developed in Section 3 are investigated in this subsection.

By linearity, the Kalman filter in (10) can be divided into two parts with ${\hat{z}}_{i}=:g_{i}+e_{i}$ as in

(28a) $$\begin{array}{cc} g_{i}\left(k+1|k+1\right) & :=g_{i}\left(k+1|k\right)+K_{i}\left(k+1\right)\left(y_{i}\left(k+1\right)-t_{i}g_{i}\left(k+1|k\right)\right), \end{array}$$

(28b) $$\begin{array}{cc} e_{i}\left(k+1|k+1\right) & :=e_{i}\left(k+1|k\right)+K_{i}\left(k+1\right)\left(a_{i}\left(k+1\right)-t_{i}e_{i}\left(k+1|k\right)\right), \end{array}$$

(28c) $$\begin{array}{cc} g_{i}\left(k+1|k\right) & :=S_{i}g_{i}\left(k|k\right)+Z_{i}^{⊤}Bu\left(k\right), \end{array}$$

(28d) $$\begin{array}{cc} e_{i}\left(k+1|k\right) & :=S_{i}e_{i}\left(k|k\right). \end{array}$$

Note that $g_{i}\left(k+1|k+1\right)$ and $e_{i}\left(k+1|k+1\right)$ have the same dynamics with (10a), while the incoming signal $y_{i}^{a}\left(k+1\right)$ is divided into two parts with $y_{i}\left(k+1\right)$ and $a_{i}\left(k+1\right)$ assigned to the dynamics of $g_{i}\left(k+1|k+1\right)$ and $e_{i}\left(k+1|k+1\right)$, respectively. Similarly, $g_{i}\left(k+1|k\right)$ and $e_{i}\left(k+1|k\right)$ have the same dynamics with (10b), whereas the incoming signal u(k) is solely assigned to the dynamics of $g_{i}\left(k+1|k\right)$. By setting the initial conditions as

$$g_{i}\left(0|0\right)={\hat{z}}_{i}\left(0|0\right)=Z_{i}^{⊤}{\bar{x}}_{0}\;\;\;\mathrm{and}\;\;\;e_{i}\left(0|0\right)=0_{\mu_{i}\times 1},$$

it easily follows from (10a) and (10b) that

(29) $$\begin{array}{c} \begin{array}{cc} {\hat{z}}_{i}\left(k+1|k+1\right) & =g_{i}\left(k+1|k+1\right)+e_{i}\left(k+1|k+1\right),\\ {\hat{z}}_{i}\left(k+1|k\right) & =g_{i}\left(k+1|k\right)+e_{i}\left(k+1|k\right). \end{array} \end{array}$$

Finally, the local observer $O_{i}$ in (9) is divided into $O_{i}^{y}$ and $O_{i}^{a}$, as follows:

(30a) $$\begin{array}{c} \begin{array}{cc} \;\;\;O_{i}^{y}:\;g_{i}\left(k+1|k+1\right) & =\left(I-K_{i}\left(k+1\right)t_{i}\right)\left(S_{i}g_{i}\left(k|k\right)+Z_{i}^{⊤}Bu\left(k\right)\right)+K_{i}\left(k+1\right)y_{i}\left(k+1\right),\;\;\; \end{array} \end{array}$$

(30b) $$\begin{array}{c} \begin{array}{cc} \;\;\;O_{i}^{a}:\;e_{i}\left(k+1|k+1\right) & =\left(I-K_{i}\left(k+1\right)t_{i}\right)S_{i}e_{i}\left(k|k\right)+K_{i}\left(k+1\right)a_{i}\left(k+1\right). \end{array} \end{array}$$

Now, define the attack-free estimation error

(31) $$\begin{array}{c} \begin{array}{cc} v_{i}\left(k+1|k+1\right) & :=g_{i}\left(k+1|k+1\right)-z_{i}\left(k+1\right),\\ v_{i}\left(k+1|k\right) & :=g_{i}\left(k+1|k\right)-z_{i}\left(k+1\right), \end{array} \end{array}$$

and we have that

(32a) $$\begin{array}{cc} v_{i}\left(k+1|k\right) & =\left(S_{i}g_{i}\left(k|k\right)+Z_{i}^{⊤}Bu\left(k\right)\right)-\left(S_{i}z_{i}\left(k\right)+Z_{i}^{⊤}Bu\left(k\right)+Z_{i}^{⊤}d\left(k\right)\right)\\ \; & =S_{i}v_{i}\left(k|k\right)-Z_{i}^{⊤}d\left(k\right) \end{array}$$

(32b)vi(k+1|k+1)=gi(k+1|k)+Ki(k+1)(yi(k+1)−tigi(k+1|k))−zi(k+1)=(I−Ki(k+1)ti)vi(k+1|k)+Ki(k+1)ni(k+1)=(I−Ki(k+1)ti)Sivi(k|k)−(I−Ki(k+1)ti)Zi⊤d(k)(32c)        +Ki(k+1)ni(k+1),

which is the same as (14) and (15) with $n_{i}^{a}$ replaced by $n_{i}$. By (29) and (31), the total state-estimation error defined in (13) satisfies

(33) $${\tilde{z}}_{i}\left(k+1|k+1\right)=v_{i}\left(k+1|k+1\right)+e_{i}\left(k+1|k+1\right),$$

and, from (30b) and (32c), its dynamic equation is given as follows:

(34) $$\begin{array}{c} \begin{array}{cc} F_{i}:\;{\tilde{z}}_{i}\left(k+1|k+1\right) & =\left(I-K_{i}\left(k+1\right)t_{i}\right)S_{i}{\tilde{z}}_{i}\left(k|k\right)-\left(I-K_{i}\left(k+1\right)t_{i}\right)Z_{i}^{⊤}d\left(k\right)\\ \; & \;\;\;+K_{i}\left(k+1\right)n_{i}\left(k+1\right)+K_{i}\left(k+1\right)a_{i}\left(k+1\right), \end{array} \end{array}$$

which is a rewrite of (15) using the fact $n_{i}^{a}=n_{i}+a_{i}$.

For notational simplicity, ${\hat{z}}_{i}\left(k|k\right)$, $v_{i}\left(k|k\right)$, and $e_{i}\left(k|k\right)$ are denoted by ${\hat{z}}_{i}\left(k\right)$, $v_{i}\left(k\right)$, and $e_{i}\left(k\right)$, respectively. Then, Equation (19) becomes

(35) $$\left[\begin{array}{c} {\hat{z}}_{1}\left(k\right)\\ \vdots \\ {\hat{z}}_{p}\left(k\right) \end{array}\right]=\left[\begin{array}{c} {Z_{1}}^{⊤}\\ \vdots \\ {Z_{p}}^{⊤} \end{array}\right]x\left(k\right)+\left[\begin{array}{c} v_{1}\left(k\right)\\ \vdots \\ v_{p}\left(k\right) \end{array}\right]+\left[\begin{array}{c} e_{1}\left(k\right)\\ \vdots \\ e_{p}\left(k\right) \end{array}\right],$$

which can be written in a compact form as

(36) $$\begin{array}{c} \hat{z}\left(k\right)=\Phi x\left(k\right)+v\left(k\right)+e\left(k\right)\in R^{\mu }. \end{array}$$

The above Equation (36) is nothing but (20) with $v^{a}$ replaced by v+e. The properties of *v* are exactly identical with those of $v^{a}$ in (22) because the derivation in (22) is under the assumption of a≡0 meaning e≡0 in this case. Thus, we have

(37) $$v\left(k\right)∼N\left(0_{\mu \times 1},P\left(k\right)\right),$$

where P(k) simply denotes P(k|k) in (23). The attack-induced signal $e\left(k\right)={\left[e_{1}^{⊤}\left(k\right),\cdots ,e_{p}^{⊤}\left(k\right)\right]}^{⊤}$ evolves according to Equation (30b) (or equivalently (28b) and (28d)) with an initial value of $e_{i}\left(0\right)=e_{i}\left(0|0\right)=0_{\mu_{i}\times 1}$. Therefore, we have $e_{i}\equiv 0_{\mu_{i}\times 1}$ for the healthy sensor with $a_{i}\equiv 0$, while $e_{i}≢0_{\mu_{i}\times 1}$ generally holds for the attacked sensor with $a_{i}≢0$. Finally, the stacked error vector $e\in R^{\mu }$ partitioned by the sequence $\left\{\mu_{i}\right\}$, is ($\left\{\mu_{i}\right\}$-stacked) q-sparse by Assumption 2.

### 4.2. Detection of Sparse Sensor Attack

In the previous subsection, the measurement data have the form $\hat{z}=\Phi x+v+e\in R^{\mu }$ with unknown signals *x*, *v*, and *e* where the noise-induced signal *v* can be considered as a random variable whose distribution is $N(0_{\mu \times 1},P)$ and the attack-induced signal *e* is ($\left\{\mu_{i}\right\}$-stacked) q-sparse. To investigate the properties of the matrix Φ in the measurement data, we borrow the definition of ($\left\{\mu_{i}\right\}$-stacked) q-error detectability and its characterization from [15]. There is a slight modification in the following Definition 1 and Lemma 1 from [15]. They do not append any additional zeros, whereas [15] adds additional zeros to match the size of all partitioned vectors and matrices.

**Definition** **1** ([15], Definition 1)**.** *For a finite sequence $\left\{\mu_{i}\right\}=\left\{\mu_{1},\mu_{2},\cdots ,\mu_{p}\right\}$ with $\mu =\sum_{i=1}^{p}\mu_{i}$, a coding matrix $\Phi \in R^{\mu \times n}$ is said to be* ($\left\{\mu_{i}\right\}$-stacked) q-error detectable  *if, for all $x,x^{\prime }\in R^{n}$ and ($\left\{\mu_{i}\right\}$-stacked) q-sparse $e\in R^{\mu }$ such that $\Phi x+e=\Phi x^{\prime }$, it holds that $x=x^{\prime }$*.

Accordingly, the matrix $\Phi \in R^{\mu \times n}$ is not ($\left\{\mu_{i}\right\}$-stacked) q-error detectable if and only if there exist $x,x^{\prime }\in R^{n}$ satisfying $x\neq x^{\prime }$, and ($\left\{\mu_{i}\right\}$-stacked) q-sparse $e\in R^{\mu }$ such that $\Phi x+e=\Phi x^{\prime }$. In other words, the matrix $\Phi \in R^{\mu \times n}$ is ($\left\{\mu_{i}\right\}$-stacked) q-error undetectable if and only if there exist a non-zero $x_{e}\in R^{n}$ and ($\left\{\mu_{i}\right\}$-stacked) q-sparse $e\in R^{\mu }$ such that $\Phi x_{e}=e$. Typically, in terms of vectors, the vector $e\in R^{\mu }$ is said to be undetectable with respect to $\Phi \in R^{\mu \times n}$ if $e=\Phi x_{e}\in R^{\mu }$ for some $x_{e}\in R^{n}$.

**Lemma** **1** ([15], Proposition 1)**.** *For a finite sequence $\left\{\mu_{i}\right\}=\left\{\mu_{1},\mu_{2},\cdots ,\mu_{p}\right\}$ with $\mu =\sum_{i=1}^{p}\mu_{i}$ and a matrix $\Phi \in R^{\mu \times n}$, the followings are equivalent:*

*(i)*  *The matrix $\Phi \in R^{\mu \times n}$ is ($\left\{\mu_{i}\right\}$-stacked) q-error detectable.*
*(ii)*  *For every set J⊂[p] satisfying |J|≥p−q, $\Phi_{I_{J}^{\left\{\mu_{i}\right\}}}$ has full column rank.*
*(iii)*  * For any $x\in R^{n}$ where $x\neq 0_{n\times 1}$, the vector $\Phi x\in R^{\mu }$ is not ($\left\{\mu_{i}\right\}$-stacked) q-sparse.*

With the estimate $\hat{x}$ of *x* obtained by MVUE of (24) in Theorem 1, we can calculate the estimated output $\Phi \hat{x}$ and generate a residual signal *r*, which is a difference between the real measurement and the estimated output, that is, $r:=\hat{z}-\Phi \hat{x}$. Then, the residual *r* becomes another random variable whose distribution is also Gaussian. Finally, the mean and covariance of the Gaussian distributed random variable *r* is computed in the following theorem.

**Theorem** **2.** *For the measurement $\hat{z}=\Phi x+v+e\in R^{\mu }$ where $\Phi \in R^{\mu \times n}$ has full column rank and v satisfies $v∼N(0_{\mu \times 1},P)$ with P>0, let $\hat{x}=\Psi \hat{z}={\left(\Phi^{⊤}P^{-1}\Phi \right)}^{-1}\Phi^{⊤}P^{-1}\hat{z}$ and*

(38) $$\begin{array}{c} \begin{array}{c} r:=\hat{z}-\Phi \hat{x}=\left(I_{\mu \times \mu }-\Phi \Psi \right)\hat{z}=\left(I_{\mu \times \mu }-\Phi {\left(\Phi^{⊤}P^{-1}\Phi \right)}^{-1}\Phi^{⊤}P^{-1}\right)\hat{z}, \end{array} \end{array}$$

*where $\Psi :={\left(\Phi^{⊤}P^{-1}\Phi \right)}^{-1}\Phi^{⊤}P^{-1}$. Then, the residual r is Gaussian distributed with mean $(I_{\mu \times \mu }-\Phi \Psi )e$ and covariance $(I_{\mu \times \mu }-\Phi \Psi )P$*,

(39) $$r∼N\left(\left(I_{\mu \times \mu }-\Phi {\left(\Phi^{⊤}P^{-1}\Phi \right)}^{-1}\Phi^{⊤}P^{-1}\right)e,P-\Phi {\left(\Phi^{⊤}P^{-1}\Phi \right)}^{-1}\Phi^{⊤}\right).$$

*Furthermore, $e=\Phi x_{e}\in R^{\mu }$ for some $x_{e}\in R^{n}$ if and only if the mean of r, $E\left[r\right](=\left(I_{\mu \times \mu }-\Phi \Psi \right)e)$, satisfies $E\left[r\right]=0_{\mu \times 1}$. In other words, e is undetectable with respect to* Φ *if and only if $E\left[r\right]=0_{\mu \times 1}$*.

**Proof.**  First, the mean of *r* is computed as follows.

(40) $$\begin{array}{c} \begin{array}{cc} E[r] & =E[\left(I_{\mu \times \mu }-\Phi {\left(\Phi^{⊤}P^{-1}\Phi \right)}^{-1}\Phi^{⊤}P^{-1}\right)\hat{z}]\\ & =\left(I_{\mu \times \mu }-\Phi {\left(\Phi^{⊤}P^{-1}\Phi \right)}^{-1}\Phi^{⊤}P^{-1}\right)E\left[\Phi x+v+e\right]\\ & =\left(I_{\mu \times \mu }-\Phi {\left(\Phi^{⊤}P^{-1}\Phi \right)}^{-1}\Phi^{⊤}P^{-1}\right)\left(\Phi x+e\right)\\ & =\left(I_{\mu \times \mu }-\Phi {\left(\Phi^{⊤}P^{-1}\Phi \right)}^{-1}\Phi^{⊤}P^{-1}\right)e=\left(I_{\mu \times \mu }-\Phi \Psi \right)e \end{array} \end{array}$$

Second, because it easily follows that

$$\begin{array}{cc} r-E[r] & =\left(I_{\mu \times \mu }-\Phi {\left(\Phi^{⊤}P^{-1}\Phi \right)}^{-1}\Phi^{⊤}P^{-1}\right)\left(\hat{z}-e\right)\\ \; & =\left(I_{\mu \times \mu }-\Phi {\left(\Phi^{⊤}P^{-1}\Phi \right)}^{-1}\Phi^{⊤}P^{-1}\right)\left(\Phi x+v\right)\\ \; & =\left(I_{\mu \times \mu }-\Phi {\left(\Phi^{⊤}P^{-1}\Phi \right)}^{-1}\Phi^{⊤}P^{-1}\right)v=\left(I_{\mu \times \mu }-\Phi \Psi \right)v, \end{array}$$

the covariance matrix is calculated as

$$\begin{array}{cc} \; & \;\;E\left[\left(r-E\left[r\right]\right){\left(r-E\left[r\right]\right)}^{⊤}\right]=E\left[\left(I_{\mu \times \mu }-\Phi \Psi \right)vv^{⊤}{\left(I_{\mu \times \mu }-\Phi \Psi \right)}^{⊤}\right]\\ \; & =\left(I_{\mu \times \mu }-\Phi \Psi \right)E\left[vv^{⊤}\right]{\left(I_{\mu \times \mu }-\Phi \Psi \right)}^{⊤}=\left(I_{\mu \times \mu }-\Phi \Psi \right)P{\left(I_{\mu \times \mu }-\Phi \Psi \right)}^{⊤}\\ \; & =\left(I_{\mu \times \mu }-\Phi {\left(\Phi^{⊤}P^{-1}\Phi \right)}^{-1}\Phi^{⊤}P^{-1}\right)P{\left(I_{\mu \times \mu }-\Phi {\left(\Phi^{⊤}P^{-1}\Phi \right)}^{-1}\Phi^{⊤}P^{-1}\right)}^{⊤}\\ \; & =P-\Phi {\left(\Phi^{⊤}P^{-1}\Phi \right)}^{-1}\Phi^{⊤}=\left(I_{\mu \times \mu }-\Phi \Psi \right)P. \end{array}$$

Moreover, note that

$$\begin{array}{cc} E[r] & =\left(I_{\mu \times \mu }-\Phi {\left(\Phi^{⊤}P^{-1}\Phi \right)}^{-1}\Phi^{⊤}P^{-1}\right)E\left[\hat{z}\right] \end{array}$$

because of (40), and

$$\begin{array}{cc} E[\hat{z}] & =E[\Phi x+v+e]=\Phi x+e. \end{array}$$

Since $\Phi {\left(\Phi^{⊤}P^{-1}\Phi \right)}^{-1}\Phi^{⊤}P^{-1}$ is a projection matrix and it projects $E[\hat{z}]$ onto the range space of Φ, R(Φ), we have $E\left[\hat{z}\right]=\Phi x+e\notin R\left(\Phi \right)$ if and only if $E\left[\hat{z}\right]\neq \Phi {\left(\Phi^{⊤}P^{-1}\Phi \right)}^{-1}\Phi^{⊤}P^{-1}E\left[\hat{z}\right]$. This implies that e∉R(Φ) if and only if $\left(I_{\mu \times \mu }-\Phi {\left(\Phi^{⊤}P^{-1}\Phi \right)}^{-1}\Phi^{⊤}P^{-1}\right)E\left[\hat{z}\right]\neq 0_{\mu \times 1}$. This completes the proof.    □

Theorem 2 clarifies the mean and covariance of the Gaussian random variable *r*, and further, characterization of undetectable attacks with statistical analysis is also given. Now, one can derive a detection criterion of ($\left\{\mu_{i}\right\}$-stacked) q-sparse errors based on the property of the residual signal *r*, assuming that $\Phi \in R^{\mu \times n}$ is ($\left\{\mu_{i}\right\}$-stacked) q-error detectable and that $e\in R^{\mu }$ is actually ($\left\{\mu_{i}\right\}$-stacked) q-sparse. This detection strategy is summarized in the following theorem.

**Theorem** **3.** *For a finite sequence $\left\{\mu_{i}\right\}=\left\{\mu_{1},\mu_{2},\cdots ,\mu_{p}\right\}$ with $\mu =\sum_{i=1}^{p}\mu_{i}$ and the measurement $\hat{z}=\Phi x+v+e\in R^{\mu }$ where $\Phi \in R^{\mu \times n}$ is ($\left\{\mu_{i}\right\}$-stacked) q-error detectable, $e\in R^{\mu }$ is ($\left\{\mu_{i}\right\}$-stacked) q-sparse, and $v\in R^{\mu }$ satisfies $v∼N(0_{\mu \times 1},P)$ with P>0, let*

$$r=\hat{z}-\Phi \hat{x}=\hat{z}-\Phi \Psi \hat{z}=\left(I_{\mu \times \mu }-\Phi \Psi \right)\hat{z}=\left(I_{\mu \times \mu }-\Phi {\left(\Phi^{⊤}P^{-1}\Phi \right)}^{-1}\Phi^{⊤}P^{-1}\right)\hat{z}$$

*be given. Then, $e=0_{\mu \times 1}$ if and only if $E\left[r\right]=0_{\mu \times 1}$. Moreover, when $e=0_{\mu \times 1}$, the vector x is exactly recovered by the expectation value of $\hat{x}=\Psi \hat{z}={\left(\Phi^{⊤}P^{-1}\Phi \right)}^{-1}\Phi^{⊤}P^{-1}\hat{z}$, that is, $x=E[\hat{x}]$, which means that $\hat{x}$ is an unbiased estimate of x.*

**Proof.**  From Theorem 2, the ($\left\{\mu_{i}\right\}$-stacked) q-sparse *e* satisfies $e=\Phi x_{e}\in R^{\mu }$ for some $x_{e}\in R^{n}$ if and only if $E\left[r\right]=0_{\mu \times 1}$. However, any non-zero $e=\Phi x_{e}\in R^{\mu }$ for some $x_{e}\in R^{n}$ is not ($\left\{\mu_{i}\right\}$-stacked) q-sparse by Lemma 1. (iii) since $\Phi \in R^{\mu \times n}$ is ($\left\{\mu_{i}\right\}$-stacked) q-error detectable. Therefore, the ($\left\{\mu_{i}\right\}$-stacked) q-sparse $e=\Phi x_{e}\in R^{\mu }$ should be zero, and the result directly follows. Furthermore, the property of an unbiased estimate (with minimum variance) is easily obtained from Theorem 1.    □

From the observation of Theorems 2 and 3, the problem of detecting a non-zero ($\left\{\mu_{i}\right\}$-stacked) q-sparse error signal *e* with a ($\left\{\mu_{i}\right\}$-stacked) q-error detectable coding matrix $\Phi \in R^{\mu \times n}$ can be rephrased as: Given the residual signal *r*, which comes from the Gaussian distribution $N(E\left[r\right],P-\Phi {\left(\Phi^{⊤}P^{-1}\Phi \right)}^{-1}\Phi^{⊤})$, determine if $E\left[r\right]=0_{\mu \times 1}$ or $E\left[r\right]\neq 0_{\mu \times 1}$. Therefore, the statistical decision theory [31] is helpful in this situation. More precisely, the $\chi^{2}$ test for fault detection [22,23], which is widely used to detect unwanted error signals, such as faults or attacks, can be applied.

One can simply apply the $\chi^{2}$ test to detect the presence of error signals in the ($\left\{\mu_{i}\right\}$-stacked) measurement $\hat{z}$ given by (36), and its operating scheme is summarized in Algorithm 1. Initially, the attack detection alarm indicator *f* is set to 0, and then the residual *r* is computed according to Equation (38). Without any error signal (that is, $e=0_{\mu \times 1}$), the residual *r* follows a Gaussian distribution $N(0,P-\Phi {\left(\Phi^{⊤}P^{-1}\Phi \right)}^{-1}\Phi^{⊤})$, which is shown in (39). Now, define the standardized residual $\zeta :={\left(P-\Phi {\left(\Phi^{⊤}P^{-1}\Phi \right)}^{-1}\Phi^{⊤})\right)}^{-\frac{1}{2}}r$ whose distribution becomes $N(0_{\mu \times 1},I_{\mu \times \mu })$. Thus, the 2-norm of ζ denoted by $g:=\zeta^{⊤}\zeta$ is an observation from a random variable g, which satisfies a $\chi^{2}$ distribution with μ degrees of freedom (DOF),

$$g∼\chi_{\mu }^{2}.$$

This means that *g* cannot be far away from zero. Finally, when *g* is greater than a threshold $\Delta_{TH}$, the attack detection alarm is triggered by setting f=1. Here, $\Delta_{TH}$ is the predetermined threshold value, and it decides the probability of false alarm and the probability of error detection. For example, when the threshold $\Delta_{TH}$ is chosen such that

(41) $$\int_{0}^{\Delta_{TH}}p_{g}\left(x\right)dx=1-\delta ,$$

where $p_{g}\left(x\right)$ denotes the PDF of the $\chi_{\mu }^{2}$ distribution, the probability of false alarm becomes δ. As the probability of false alarm δ becomes smaller, the probability of error detection also decreases, which implies that there is a trade-off between the small false alarm and the high error detection ratio. Thus, one needs to choose $\Delta_{TH}$ as a good compromise between these two conflicting requirements.
**Algorithm 1**  Detection scheme based on the $\chi^{2}$ test**Input:** $\hat{z}$ **Output:** *f* **Initialization:** f=0 1: ${\hat{x}}_{\mathrm{MVUE}}={\left(\Phi^{⊤}P^{-1}\Phi \right)}^{-1}\Phi^{⊤}P^{-1}\hat{z}$ 2: $r=\hat{z}-\Phi {\hat{x}}_{\mathrm{MVUE}}$ 3: $\zeta ={\left(P-\Phi {\left(\Phi^{⊤}P^{-1}\Phi \right)}^{-1}\Phi^{⊤})\right)}^{-\frac{1}{2}}r$ 4: $g=\zeta^{⊤}\zeta$ 5: **if** $g\leq \Delta_{TH}$**then** 6:    f=0 7: **else if** 
$g>\Delta_{TH}$
**then**
 8:    f=1
 9: **end if**


### 4.3. Secure State Estimation under a Sparse Sensor Attack

In this subsection, an attack-resilient and secure state estimation scheme, which reconstructs the optimal estimate for the state *x* under Assumptions 1–3, is developed. First, characterization of the matrix Φ defined in (21) under Assumption 3 is given as follows.

**Lemma** **2** ([15], Proposition 1,2,3,6)**.** *For a finite sequence $\left\{\mu_{i}\right\}=\left\{\mu_{1},\mu_{2},\cdots ,\mu_{p}\right\}$ with $\mu_{i}=\mathrm{rank}\left(G_{i}\right)$ for i∈[p] where $G_{i}$ is the observability matrix given in (4), the followings are equivalent:*

*(i)*  *The pair (A,C) is 2q redundant observable.*
*(ii)*  *The matrix* Φ *is ($\left\{\mu_{i}\right\}$-stacked) 2q-error detectable.*
*(iii)*  * For every set J⊂[p] satisfying |J|≥p−2q, $\Phi_{I_{J}^{\left\{\mu_{i}\right\}}}$ has full column rank.*
*(iv)*  * The pair (A,C) is observable under q-sparse sensor attacks.*

Note that the redundancy for observability is 2q, which is twice the sparsity of the attack signal. This is the key point of constructing the state estimation algorithm. We can examine each subset $J_{k}\subset \left[p\right]$ of sensors whose size is p−q. In other words, we have pq number of subsets J1,J2,⋯,Jpq where $J_{k}\subset \left[p\right]$ and $|J_{k}|=p-q$ for k=1,2,⋯,pq. Since Φ is ($\left\{\mu_{i}\right\}$-stacked) 2q-error detectable by Assumption 3 and Lemma 2.(ii), it easily follows that $\Phi_{I_{J_{k}}^{\left\{\mu_{i}\right\}}}$ is q-error detectable for $J_{k}$ with $|J_{k}|=p-q$. This means that, even after removing any q sensors, the remaining outputs still have q redundancy for observability. Therefore, the detection scheme of Theorem 3, which relies on the ($\left\{\mu_{i}\right\}$-stacked) q-error detectability of the coding matrix, can be applied for each subset $J_{k}\subset \left[p\right]$ satisfying $|J_{k}|=p-q$.

**Figure 2:**
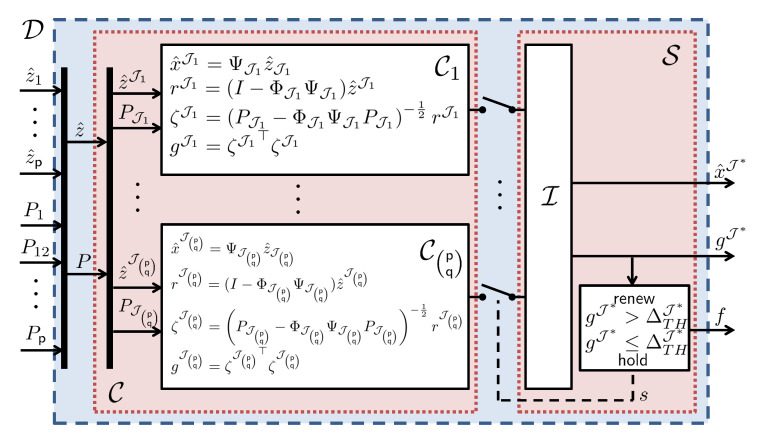
Configuration of the resilient estimation scheme with Gaussian disturbance/noise.

The configuration of the secure state estimator, which replaces the information fusion center D in Figure 1, is sketched in Figure 2, and its operation is described in Algorithm 2. Before explaining the operation, let Ψ denote ${\left(\Phi^{⊤}P^{-1}\Phi \right)}^{-1}\Phi^{⊤}P^{-1}$ where Φ and *P* are given in (21) and (23), respectively. Furthermore, the notation for a sub-matrix is slightly abused for simplicity. For example, $P_{J}$, $\Phi_{J}$, and $\Psi_{J}$ denote

$$P_{I_{J}^{\left\{\mu_{i}\right\}},\;\;I_{J}^{\left\{\mu_{i}\right\}}},\;\;\Phi_{I_{J}^{\left\{\mu_{i}\right\}}},\;\;\mathrm{and}\;\;\;{\left(\Phi_{I_{J}^{\left\{\mu_{i}\right\}}}^{⊤}{\left(P_{I_{J}^{\left\{\mu_{i}\right\}},I_{J}^{\left\{\mu_{i}\right\}}}\right)}^{-1}\Phi_{I_{J}^{\left\{\mu_{i}\right\}}}\right)}^{-1}\Phi_{I_{J}^{\left\{\mu_{i}\right\}}}^{⊤}{\left(P_{I_{J}^{\left\{\mu_{i}\right\}},I_{J}^{\left\{\mu_{i}\right\}}}\right)}^{-1},$$

respectively, where $I_{J}^{\left\{\mu_{i}\right\}}:={⋃}_{j\in J}^{}\left\{\left(\sum_{i=1}^{j-1}\mu_{i}\right)+1,\left(\sum_{i=1}^{j-1}\mu_{i}\right)+2,\cdots ,\sum_{i=1}^{j}\mu_{i}\right\}$. Recall that $P_{I_{J}^{\left\{\mu_{i}\right\}},I_{J}^{\left\{\mu_{i}\right\}}}$ denotes the matrix obtained from *P* by eliminating all i-th rows and all j-th columns such that $i\notin I_{J}^{\left\{\mu_{i}\right\}}$ and $j\notin I_{J}^{\left\{\mu_{i}\right\}}$.

Initially, an attack-free index set $J^{*}$, a state estimate $\hat{x}$, a standardized residual’s norm *g*, and a fault alarm signal *f*, are set to [p], $\Psi \hat{z}$, 0, and 0, respectively. The algorithm continually checks if there is any attack in the index set $J^{*}$ based on Algorithm 1. For the given index set $J^{*}$, the algorithm essentially calculates the MVUE $\hat{x}=\Psi_{J^{*}}{\hat{z}}_{J^{*}}$, the residual $r={\hat{z}}_{J^{*}}-\Phi_{J^{*}}\hat{x}$, the standardized residual $\zeta ={\left(P_{J^{*}}-\Phi_{J^{*}}\Psi_{J^{*}}P_{J^{*}}\right)}^{-\frac{1}{2}}r$, and its 2-norm $g=\zeta^{⊤}\zeta$ only with the measurement and covariance data from the subset $J^{*}\subset \left[p\right]$. Recall from Theorem 2 that, if $e_{j}=e_{I_{j}^{\left\{\mu_{i}\right\}}}=0_{\mu_{j}\times 1}$ for all $j\in J^{*}$, we have $r∼N(0_{\mu_{J^{*}}\times 1},P_{J^{*}}-\Phi_{J^{*}}\Psi_{J^{*}}P_{J^{*}})$ where $\mu_{J^{*}}:=\sum_{j\in J^{*}}\mu_{j}=\left|I_{J^{*}}^{\left\{\mu_{i}\right\}}\right|$, and thus, $g=\zeta^{⊤}\zeta$ is an observation from a random variable $g_{J^{*}}$, which satisfies a $\chi^{2}$ distribution with $\mu_{J^{*}}$ DOF,

(42) $$g_{J^{*}}∼\chi_{\mu_{J^{*}}}^{2}.$$

Therefore, *g* is used to detect the presence of attack in the subset $J^{*}$ by the $\chi^{2}$ test. We compare *g* with the threshold $\Delta_{TH}^{J^{*}}$, which is designed before running the algorithm and determines the probability of false alarm and the probability of error detection. If $g\leq \Delta_{TH}^{J^{*}}$, the index set $J^{*}$ is declared to be attack-free by setting f=0 and the algorithm simply maintains the selected optimal index set $J^{*}$. Otherwise, when *g* is greater than the threshold $\Delta_{TH}^{J^{*}}$, the attack detection alarm is triggered by setting f=1, and the algorithm starts the process of searching new attack-free index set.
**Algorithm 2** Operation of the resilient estimation with Gaussian disturbance/noise**Input:** ${\hat{z}}_{1}$, ${\hat{z}}_{2}$, ⋯, ${\hat{z}}_{p}$, $P_{1}$, $P_{12}$, ⋯, $P_{p(p-1)}$, $P_{p}$ **Output:** $J^{*}$, $\hat{x}$, *g*, *f* **Initialization:** $J^{*}=\left[p\right]$, $\hat{x}=\Psi \hat{z}$, g=0, f=0 1: **while** system (1) is running **do**
 2:    $\hat{x}=\Psi_{J^{*}}{\hat{z}}_{J^{*}}$
 3:    $r={\hat{z}}_{J^{*}}-\Phi_{J^{*}}\hat{x}$
 4:    $\zeta ={\left(P_{J^{*}}-\Phi_{J^{*}}\Psi_{J^{*}}P_{J^{*}}\right)}^{-\frac{1}{2}}r$
 5:    $g=\zeta^{⊤}\zeta$
 6:    **if** $g\leq \Delta_{TH}^{J^{*}}$ **then**
 7:       f=0
 8:    **else if** $g>\Delta_{TH}^{J^{*}}$ **then**
 9:       f=1
 10:       **for** J⊂[p] satisfying |J|=p−q **do**
 11:          ${\hat{x}}^{J}=\Psi_{J}{\hat{z}}_{J}$
 12:          $r^{J}={\hat{z}}_{J}-\Phi_{J}{\hat{x}}^{J}$
 13:          $\zeta^{J}={\left(P_{J}-\Phi_{J}\Psi_{J}P_{J}\right)}^{-\frac{1}{2}}r^{J}$
 14:          $g^{J}={\zeta^{J}}^{⊤}\zeta^{J}$
 15:       **end for**
 16:       $J^{*}=\underset{\begin{array}{c} J\subset [p]\\ |J|=p-q \end{array}}{arg max}\;p_{g_{J}}\left(g^{J}\right)$
 17:    **end if**
 18: **end while**


In order to find a new attack-free index set and, consequently, to recover the state *x* from the new index set, we search all subsets $J_{k}$’s in [p] whose size is p−q. For a detailed explanation, let

J1,J2,⋯,Jpq

be the set $\left\{J\subset [p]:\;|J|=p-q\right\}$. For each subset $J_{k}$ where k∈pq, the computing module $C_{k}$ calculates the MVUE ${\hat{x}}^{J_{k}}=\Psi_{J_{k}}{\hat{z}}_{J_{k}}$, the residual $r^{J_{k}}={\hat{z}}_{J_{k}}-\Phi_{J_{k}}{\hat{x}}^{J_{k}}$, the standardized residual $\zeta^{J_{k}}={\left(P_{J_{k}}-\Phi_{J_{k}}\Psi_{J_{k}}P_{J_{k}}\right)}^{-\frac{1}{2}}r^{J_{k}}$, and its 2-norm $g^{J_{k}}={\zeta^{J_{k}}}^{⊤}\zeta^{J_{k}}$ only with the measurement and covariance data from the subset $J_{k}$. Now, the new optimal subset $J^{*}$ is decided by the maximum likelihood (ML) decision rule with the values of $g^{J_{k}}$’s, and the selector S chooses the optimal index set $J^{*}$ by the ML decision rule. To this end, we wish to distinguish between pq hypotheses, H1,H2,⋯,Hpq, which are given as follows:

$$H_{k}:\;\mathrm{the}\;\mathrm{set}\;J_{k}\;\mathrm{is}\;\mathrm{attack}-\mathrm{free},\;i.e.,\;e_{j}=e_{I_{j}^{\left\{\mu_{i}\right\}}}=0_{\mu_{j}\times 1}\;\mathrm{for}\;\mathrm{all}\;j\in J_{k}.$$

Let us denote $g_{k}$ as a random variable such that $g^{J_{k}}$ is a single observation from $g_{k}$, whereas $g_{J_{k}}$ denotes a random variable such that

$$g_{J_{k}}∼\chi_{\mu_{J_{k}}}^{2}$$

with $\mu_{J_{k}}:=\sum_{j\in J_{k}}\mu_{j}=\left|I_{J_{k}}^{\left\{\mu_{i}\right\}}\right|$ and $p_{g_{J_{k}}}$ is the PDF of the $\chi_{\mu_{J_{k}}}^{2}$ distribution. Note that, if the sensors indexed by $J_{k}$ are attack-free, then the random variable $g_{k}$ as well as $g_{J_{k}}$ follows the $\chi^{2}$ distribution with $\mu_{J_{k}}$ DOF. The ML decision rule choose the hypothesis $H_{k^{*}}$ and the corresponding optimal index set $J_{k^{*}}$ that maximize the likelihood $p_{g_{k}}\left(g^{J_{k}};H_{k}\right)$, which is the PDF of $g_{k}$ being equal to the observation $g^{J_{k}}$ under the hypothesis $H_{k}$ (that is, under the condition that there is no attack signal in the measurements indexed by $J_{k}$). Therefore, we have

J*=Jk*=arg maxk∈pqpgkgJk;Hk=arg maxJ⊂[p]|J|=p−qpgJgJ,

where the last equality comes from the fact that $g_{k}∼\chi_{\mu_{J_{k}}}^{2}$ under the hypothesis $H_{k}$ so that it follows the PDF of the $\chi^{2}$ distribution. Therefore, from the index set $J_{k^{*}}$ corresponding to the ML hypothesis $H_{k^{*}}$, the MVUE of the newly selected optimal index set $J^{*}\left(=J_{k^{*}}\right)$, ${\hat{x}}^{J^{*}}$, becomes the final suboptimal estimate of *x*.

**Remark** **2.** The proposed algorithm selects the subset of sensors $J^{*}\subset \left[p\right]$, which is most likely to be attack-free with $|J^{*}|=p-q$. Moreover, if the selected set $J^{*}$ is actually attack-free, it gives the minimum variance with unbiased estimation. In short, Algorithm 2 generates a state estimate, which is most likely to have minimum variance with unbiased mean. However, we say that it is a suboptimal estimate of x instead of the optimal estimate because the decentralized multi-sensor information fusion Kalman filter cannot ensure to achieve the centralized optimal covariance even without attack as illustrated in ([24], Section 5).

**Remark** **3.** *Note that Algorithm 2 needs to prepare pq candidates and compare all those candidates. The time complexity of the error correction algorithm depends on the number of combinations pq, and thus, it has the polynomial time complexity of $O(p^{\min\{q,\;p-q\}})$. Therefore, the proposed algorithm may not be scalable for very large p with q≈p/2 due to the combinatorial nature of the algorithm. The time complexity could be reduced by imposing additional restrictive assumptions as done in [20,21] which reformulate the problem into a convex optimization problem. However, in our scheme demanding minimal assumptions, the comibinatorial algorithm only needs to operate when an attack is detected. In addition, most of the time, only the attack detection algorithm requiring a small amount of computation, is executed. Another advantage of the proposed algorithm is that its space complexity is linear with the number of sensors p, that is, O(p). The total memory size required for local observers is $\sum_{i=1}^{p}\mu_{i}\leq np$, whereas if all possible combinations of estimator candidates are configured as real observers, the observer’s size becomes npq.*

## 5. Simulation Results

**Figure 3:**
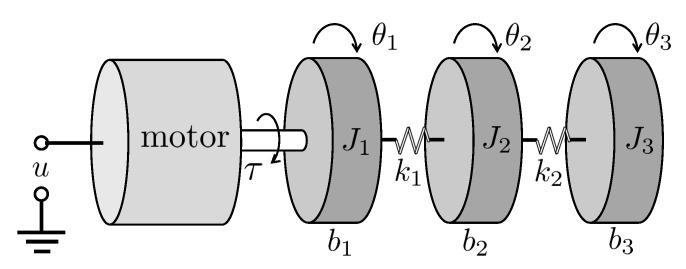
Motor control system of multi-DOF torsion modules.

We consider a motor-controlled multi-DOF torsion system [34] as depicted in Figure 3. A continuous-time state-space model of the system when the control input is the torque τ (N·m) generated by the servo motor is given by

(43) $$\begin{array}{c} P_{c}^{\prime }:\left\{\begin{array}{c} \dot{x}\left(t\right)=A_{c}^{\prime }x\left(t\right)+B_{c}^{\prime }\tau \left(t\right)+d\left(t\right)\\ y\left(t\right)=C_{c}x\left(t\right)+n\left(t\right) \end{array}\right. \end{array}$$

with the matrices

(44) $$\begin{array}{c} \begin{array}{cc} A_{c}^{\prime } & =\left[\begin{array}{cccccc} 0 & 1 & 0 & 0 & 0 & 0\\ -\frac{k_{1}}{J_{1}} & -\frac{b_{1}}{J_{1}} & \frac{k_{1}}{J_{1}} & 0 & 0 & 0\\ 0 & 0 & 0 & 1 & 0 & 0\\ \frac{k_{1}}{J_{2}} & 0 & -\frac{k_{1}+k_{2}}{J_{2}} & -\frac{b_{2}}{J_{2}} & \frac{k_{2}}{J_{2}} & 0\\ 0 & 0 & 0 & 0 & 0 & 1\\ 0 & 0 & \frac{k_{2}}{J_{3}} & 0 & -\frac{k_{2}}{J_{3}} & -\frac{b_{3}}{J_{3}} \end{array}\right],\;B_{c}^{\prime }=\left[\begin{array}{c} 0\\ \frac{1}{J_{1}}\\ 0\\ 0\\ 0\\ 0 \end{array}\right],\\ C_{c} & =\left[\begin{array}{cccccc} 1 & 0 & 0 & 0 & 0 & 0\\ 0 & 0 & 1 & 0 & 0 & 0\\ 0 & 0 & 0 & 0 & 1 & 0\\ 1 & 0 & -1 & 0 & 0 & 0\\ 0 & 0 & 1 & 0 & -1 & 0 \end{array}\right], \end{array} \end{array}$$

where

$$\begin{array}{c} x:=\left[\begin{array}{c} \theta_{1}\\ {\dot{\theta }}_{1}\\ \theta_{2}\\ {\dot{\theta }}_{2}\\ \theta_{3}\\ {\dot{\theta }}_{3} \end{array}\right]\;\mathrm{and}\;\;y:=\left[\begin{array}{c} \theta_{1}\\ \theta_{2}\\ \theta_{3}\\ \theta_{1}-\theta_{2}\\ \theta_{2}-\theta_{3} \end{array}\right] \end{array}$$

are the state variable and the output measurement, respectively. Here, the unit for angular positions θ’s and the unit for angular velocities $\dot{\theta }$’s are (rad) and (rad/s), respectively. The parameters are borrowed from [34], and we have that $J_{1}=0.0022$, $J_{2}=J_{3}=0.000545$ ($\mathrm{kg}\;\cdot \;m^{2}$) for the moment of inertia, $b_{1}=0.015$, $b_{2}=b_{3}=0.0015$ (N·m/(rad/s)) for the viscous damping ratio, and $k_{1}=k_{2}=1$ (N·m/rad) for the flexible stiffness.

Note that the dynamics are the same as those of the three inertia system considered in [15]; however, Figure 3 additionally considers the servo motor system given as follows:

(45) $$\begin{array}{c} \begin{array}{c} \tau =\frac{\eta_{g}K_{g}\eta_{m}k_{t}\left(u-K_{g}k_{m}{\dot{\theta }}_{1}\right)}{R_{m}}, \end{array} \end{array}$$

which generates the torque τ (N·m) from the input voltage of *u* (V). The parameters for the servo system are also borrowed from [34], and we have that $\eta_{g}=0.9$ for the gearbox efficiency, $K_{g}=70$ for the total gear ratio, $\eta_{m}=0.69$ for the motor efficiency, $k_{t}=0.00768$ (N·m/A) for the motor current torque constant, $k_{m}=0.00768$ (V/(rad/s)) for the motor back electromotive force (EMF) constant, and $R_{m}=2.6$ (Ω) for the motor armature resistance. Thus, the final continuous-time plant with the voltage *u* (V) as an input signal is obtained as

(46) $$\begin{array}{c} P_{c}:\left\{\begin{array}{c} \dot{x}\left(t\right)=A_{c}x\left(t\right)+B_{c}u\left(t\right)+d\left(t\right)\\ y\left(t\right)=C_{c}x\left(t\right)+n\left(t\right) \end{array}\right. \end{array}$$

with the matrices

(47) $$\begin{array}{c} \begin{array}{cc} A_{c} & =\left[\begin{array}{cccccc} 0 & 1 & 0 & 0 & 0 & 0\\ -\frac{k_{1}}{J_{1}} & -\frac{b_{1}}{J_{1}}-\frac{\eta_{g}K_{g}^{2}\eta_{m}k_{t}k_{m}}{R_{m}}\frac{1}{J_{1}} & \frac{k_{1}}{J_{1}} & 0 & 0 & 0\\ 0 & 0 & 0 & 1 & 0 & 0\\ \frac{k_{1}}{J_{2}} & 0 & -\frac{k_{1}+k_{2}}{J_{2}} & -\frac{b_{2}}{J_{2}} & \frac{k_{2}}{J_{2}} & 0\\ 0 & 0 & 0 & 0 & 0 & 1\\ 0 & 0 & \frac{k_{2}}{J_{3}} & 0 & -\frac{k_{2}}{J_{3}} & -\frac{b_{3}}{J_{3}} \end{array}\right],\;B_{c}=\left[\begin{array}{c} 0\\ \frac{\eta_{g}K_{g}\eta_{m}k_{t}}{R_{m}}\frac{1}{J_{1}}\\ 0\\ 0\\ 0\\ 0 \end{array}\right], \end{array} \end{array}$$

and the same $C_{c}$ as in (44). Finally, the zero-order hold equivalent model of (46) is used for the discrete-time model P in (1), and the matrices are calculated by

(48) $$\begin{array}{c} A:=e^{A_{c}T_{s}},\;B:=\left(\int_{0}^{T_{s}}e^{A_{c}\tau }d\tau \right)B_{c},\;C:=C_{c} \end{array}$$

with the sampling time of $T_{s}=0.002$ (s). By examining all possible combinations of sensors, it follows that the system P in (1) with *A* and *C* given in (48) is 2-redundant observable, and hence it is observable under 1-sparse sensor attack by Lemma 2.

In addition, the disturbance *d* and the noise *n* are assumed to satisfy Assumption 1 with

$$\begin{array}{c} \begin{array}{cc} Q & =0.001^{2}\times \left[\begin{array}{cccccc} 1 & 0 & 0 & 0 & 0 & 0\\ 0 & 9 & 0 & 0 & 0 & 0\\ 0 & 0 & 1 & 0 & 0 & 0\\ 0 & 0 & 0 & 1 & 0 & 0\\ 0 & 0 & 0 & 0 & 1 & 0\\ 0 & 0 & 0 & 0 & 0 & 1 \end{array}\right],\;R=0.001^{2}\times \left[\begin{array}{ccccc} 1 & 0 & 0 & 1 & 0\\ 0 & 1 & 0 & -1 & 1\\ 0 & 0 & 1 & 0 & -1\\ 1 & -1 & 0 & 3 & -1\\ 0 & 1 & -1 & -1 & 3 \end{array}\right], \end{array} \end{array}$$

and the initial state x(0) of the system (46) satisfies $x\left(0\right)∼N\left({\bar{x}}_{0},P_{0}\right)$ as stated in Assumption 1 with the mean ${\bar{x}}_{0}$ and the covariance $P_{0}$ given by

$$\begin{array}{c} \begin{array}{cc} {\bar{x}}_{0} & =\left[\begin{array}{c} 0\\ 0\\ 0\\ 0\\ 0\\ 0 \end{array}\right],\;P_{0}=\left[\begin{array}{cccccc} 1 & 0 & 0 & 0 & 0 & 0\\ 0 & 1 & 0 & 0 & 0 & 0\\ 0 & 0 & 1 & 0 & 0 & 0\\ 0 & 0 & 0 & 1 & 0 & 0\\ 0 & 0 & 0 & 0 & 1 & 0\\ 0 & 0 & 0 & 0 & 0 & 1 \end{array}\right]. \end{array} \end{array}$$

**Figure 4:**
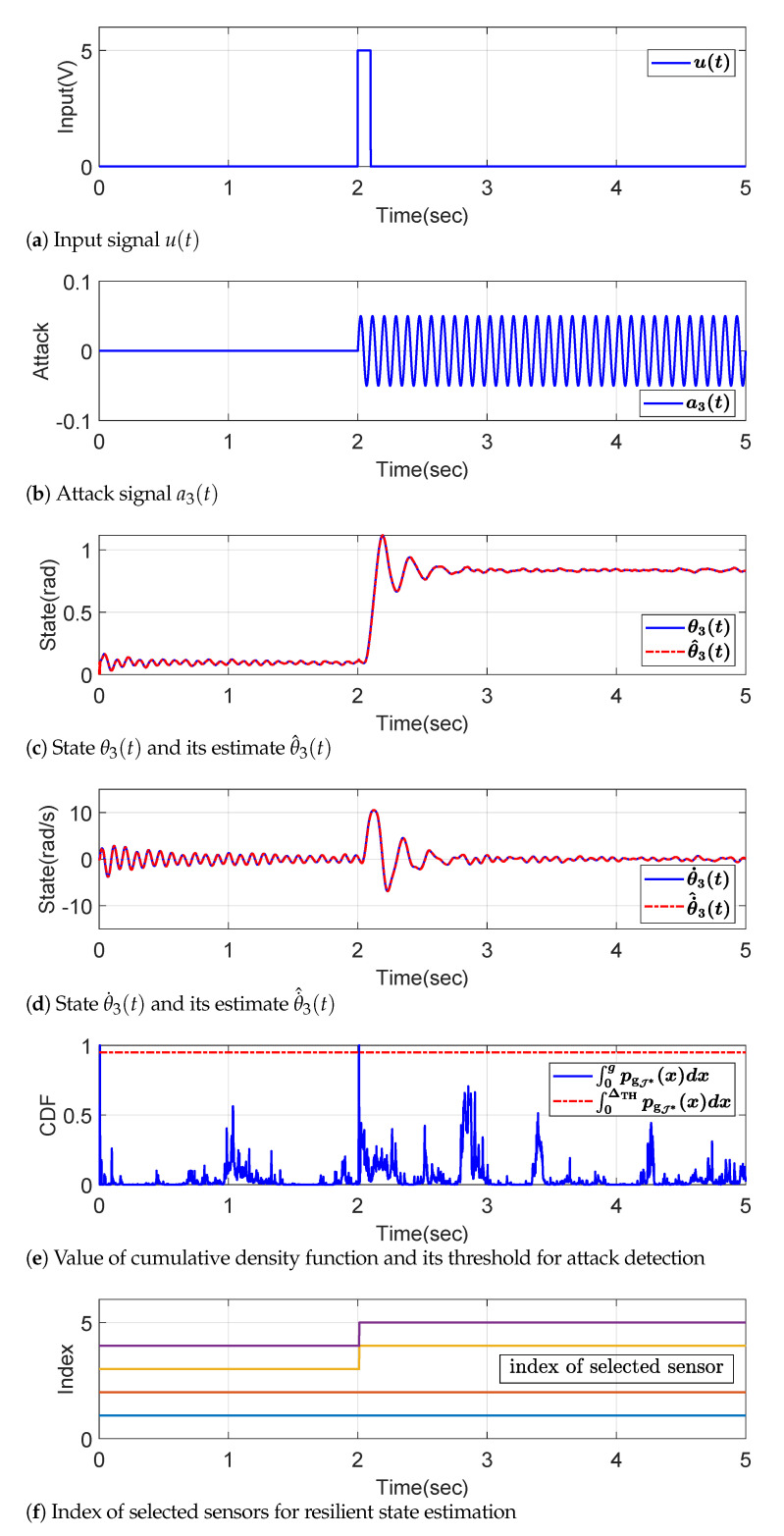
Plot of signals in a multi-DOF torsion system.

The simulation is performed under 1-sparse sensor attack on the third sensor with the signal shown in Figure 4b, which is made to mimic the motion pattern by the natural frequency as observed in Figure 4c,d. Moreover, the attack starts at 2 second, which is the same time when the square pulse input *u* is injected into the system as described in Figure 4a. Even under the attack signal, the resilient state estimation with multi-sensor information fusion Kalman filter based on the observability decomposition developed in Section 3 and Section 4 works well. The states are recovered with a small error as demonstrated in Figure 4c,d, which are the state estimation results for $\theta_{3}$ and ${\dot{\theta }}_{3}$, respectively.

In this simulation, the threshold $\Delta_{TH}$ for the attack detection is chosen by δ=0.05 in (41) so that the cumulative density function (CDF) satisfies $\int_{0}^{\Delta_{TH}}p_{g_{J^{*}}}\left(x\right)dx=0.95$ where $p_{g_{J^{*}}}$ is the PDF of a random variable $g_{J^{*}}$, which satisfies a $\chi^{2}$ distribution with $\mu_{J^{*}}$ DOF, as stated in (42). Since Figure 4e shows that the 2-norm of the standardized residual, *g*, exceeds the threshold $\Delta_{TH}$ at the instant of 2 second, which is the initiation time of the attack, it is judged that there is an attack (the lines from 8 to 9 in Algorithm 2) and the estimation scheme begins to search the indices of attack-free sensors (the lines from 10 to 16 in Algorithm 2). As a result of the search algorithm, a new set of sensor indices is found by the ML decision rule (the line 16 in Algorithm 2), and the attacked third senor is excluded from 2 second as depicted in Figure 4f.

## 6. Conclusions

In this paper, the multi-sensor information fusion Kalman filter proposed in [24,25] was improved using the observability decomposition to ensure the convergence of the error covariance matrix of each local observer. The local observer of a decentralized Kalman filter with only a single sensor was designed for an observable subspace instead of the entire n-dimensional state vector without any global information. Then, the proposed decentralized information fusion Kalman filter was applied to the secure state estimation problem where some of sensors were compromised by a malicious attacker.

To cope with the zero-mean Gaussian distributed disturbances/noises, a local Kalman filter replaced the partial Luenberger observer designed in [15], where bounded disturbances/noises were considered in the state estimation problem under sparse sensor attacks. When there was no attack, the proposed algorithm guaranteed an optimal state estimate in the sense of minimum variance, and it generated a state estimate that was most likely to have the minimum variance with an unbiased mean in the presence of sparse sensor attacks.

The proposed algorithm can be applied to cyber-physical systems, including complex sensor networks operating based on linear dynamics under sparse sensor attacks as well as Gaussian disturbances/noises. We imposed the minimal assumption of the redundant observability, which is known to be the equivalent condition to solve the problem. Furthermore, the computational time was alleviated by running only a relatively light attack detection scheme for most of the execution time, and the memory size of the observer was reduced by constructing local observers only for observable subspaces.

One possible direction of future research is to develop a distributed attack-resilient state estimator. While this paper proposed a decentralized Kalman filter scheme, the fusion center collects all the data from each sensors. Although the construction of local Kalman filters is decentralized, the information fusion method is still centralized. Therefore, it is necessary to develop a fully distributed attack-resilient state estimation technique for a general sensor network without any central information fusion center.

## Data Availability

Not applicable.

## Abbreviations

The following abbreviations are used in this manuscript:

LTI	Linear Time Invariant
i.i.d.	independent and identically distributed
MVUE	Minimum Variance Unbiased Estimator
BLUE	Best Linear Unbiased Estimator
PDF	Probability Density Function
DOF	Degrees Of Freedom
ML	Maximum Likelihood
EMF	ElectroMotive Force
CDF	Cumulative Density Function
